# Targeting CAR-T cell exhaustion in non-Hodgkin lymphoma: mechanistic insights and emerging clinical strategies

**DOI:** 10.3389/fimmu.2026.1727926

**Published:** 2026-04-21

**Authors:** Anirudh Rao, Christina Darwish, Kerry S. Campbell, Rashmi Khanal, Henry Fung, Anthony Stack

**Affiliations:** 1 Department of Medicine, Temple University Hospital, Philadelphia, PA, United States; 2 Department of Hematology and Oncology, Fox Chase Cancer Center, Philadelphia, PA, United States; 3 Institute for Cancer Research, Fox Chase Cancer Center, Philadelphia, PA, United States; 4 Department of Bone Marrow Transplant & Cellular Therapies, Fox Chase Cancer Center, Philadelphia, PA, United States

**Keywords:** B-cell malignancies, CAR-T, immunotherapy, non-Hodgkin lymphoma, T-cell exhaustion (Tex)

## Abstract

Although the response rates to chimeric antigen receptor T (CAR-T) therapy in patients with treatment-resistant lymphoma are high, the majority of patients relapse. CAR-T cell exhaustion, characterized by the progressive loss of T-cell effector functions due to several molecular and epigenetic pathways, is a major mediator of CAR-T cell failure. Strategies to prevent CAR-T cell exhaustion, including modifications to the CAR structure, addition of adjunctive agents, and alternative product manufacturing strategies have shown promise. In this review, we discuss the mechanisms of CAR-T cell exhaustion and describe strategies for its mitigation, with the aim of supporting further research in this critical area.

## Introduction

Chimeric antigen receptor T (CAR-T) cells are genetically engineered T lymphocytes modified to express chimeric antigen receptors (CARs), enabling precise recognition and targeting of tumor-associated antigens, such as CD19 expressed on malignant B-cells or BCMA on malignant plasma cells (1, 2). Beginning with foundational preclinical studies in the 1980s, it was demonstrated that CARs, which incorporate antibody-derived antigen recognition domains combined with T-cell receptor signaling components, allow for higher affinity and major histocompatibility complex (MHC) independent cell mediated cytotoxicity (3, 4). However, early CAR-T cell constructs were challenged by poor anti-tumor activity and *in vivo* persistence (2). As native T-cell activation typically requires two signals, a primary signal triggered by TCR engagement with the MHC and a second co-stimulatory signal that enhances proliferation and persistence, it was hypothesized that adding this second signal would enhance CAR-T cell immune response (5). Second generation CAR-T cells which added co-stimulatory domains, CD28 or 4-1BB, to the endodomain were thus developed and showed success in improving cytotoxicity and persistence (6, 7).

To date, four “second generation” CAR-T products based on the two-signal platform have been approved by the US Food and Drug Administration for various non-Hodgkin lymphomas: brexucabtagene autoleucel (brexu-cel), axicabtagene ciloleucel (axi-cel), lisocabtagene maraleucel (liso-cel) and tisagenlecleucel (tisa-cel) (**Table 1**) (1, 8–20, 22, 23). These four products share a common antigen target of CD19 and structurally differ in their co-stimulatory domains, with the former two utilizing a CD28 co-stimulatory domain, and the latter two utilizing a 4-1BB co-stimulatory domain (1). Unfortunately, despite promising response rates among patients with relapsed and refractory non-Hodgkin lymphomas, the majority of patients treated with second generation CAR-T cells do not achieve long term remission, with most relapsing within the first year of treatment (24–26). Survival for patients relapsing after CAR-T therapy remains dismal, with most succumbing to their disease within 6 months of relapse (27).

**Table 1:** CAR-T products approved for non-Hodgkin lymphomas.

Name	Target Antigen	Costimulatory Domain	Disease	Initial FDA Approval Date	Pivotal Studies	ORR	CR	PFS	OS
Tisagenlecleucel	CD19	4-1BB	LBCL	May 1, 2018	JULIET (8)	53%	39%	Median PFS 2.9 months	Median OS 11.1 months
			Follicular Lymphoma	May 28, 2022	ELARA (9, 10)	86%	69%	PFS 57.4% at 2 years	OS 87.7 % at 2 years
Axicabtagene ciloleucel	CD19	CD28	LBCL	Oct 18, 2017	ZUMA-1 (11, 12) ZUMA-7 (13)	82% 83%	54% 54%	Median PFS 5.9 months Median PFS 14.7 months	Median OS 25.8 months OS 55% at 4 years
			Follicular Lymphoma	March 5, 2021	ZUMA-5 (14)	94%	79%	Median PFS 40.2 months	OS 76% at 3 years
Brexucabtagene autoleucel	CD19	CD28	Mantle Cell Lymphoma	Jul 24, 2020	ZUMA-2 (9, 10)	91%	68%	Median PFS 25.8 months	Median OS 46.6 months
Lisocabtagene maraleucel	CD19	4-1BB	LBCL	Feb 5, 2021	TRANSCEND NHL 001 (15) TRANSFORM (16, 17)	73% 80%	53% 68%	Median PFS 6.8 months PFS 51% at 36 months	Median OS 27.3 months OS 63% at 36 months
			Follicular Lymphoma	May 15, 2024	TRANSCEND-FL (18)	97%	94%	PFS 83% at 12 months	OS 93% at 12 months
			Mantle Cell Lymphoma	May 30, 2024	TRANSCEND-MCL (19)	83.1%	72.3%	Median PFS 15.3 months	Median OS 18.2 months
			CLL/SLL	June 26, 2024	TRANSCEND CLL-004 (20)	47%	18%	Median PFS 17.9 months	Median OS 43.2 months
			Marginal Zone Lymphoma	Dec 4, 2025	TRANSCEND-FL (21)	95%	62%	PFS 86% at 24 months	OS 90% at 24 months

*ORR, overall response rate; CR, complete remission; EFS, event free survival; PFS, progression free survival; OS, overall survival.

Although CD19 mutations and downregulated CD19 membrane expression appear to underlie some lymphoma relapses after CD19-directed CAR-T cell therapy, these appear to be a minority of cases and are predominantly notable in late-relapses (28, 29). Recently, both preclinical and clinical studies have identified CAR-T cellular exhaustion as a key contributor to CAR-T cell failure in multiple disease states (30). Although this self-regulating characteristic of native T-cells serves an important physiological role in limiting host damage during periods of chronic inflammation, the dynamic exhaustion resulting from tonic or repetitive stimulation of CARs likely contributes to detrimental downregulation of the CAR-T cell effector functions, thus highlighting it as an important target for therapeutic intervention (31). In this review, we summarize the mechanisms underlying CAR-T cell exhaustion with a focus on those mechanisms relevant to lymphoma. We then discuss ongoing and proposed strategies to overcome this challenge, specifically highlighting novel efforts to optimize the CAR construct, adjunctive treatments for improving CAR-T efficacy and manufacturing strategies focused on improving CAR-T cell quality.

## Native T-cell exhaustion

**Figure 1:**
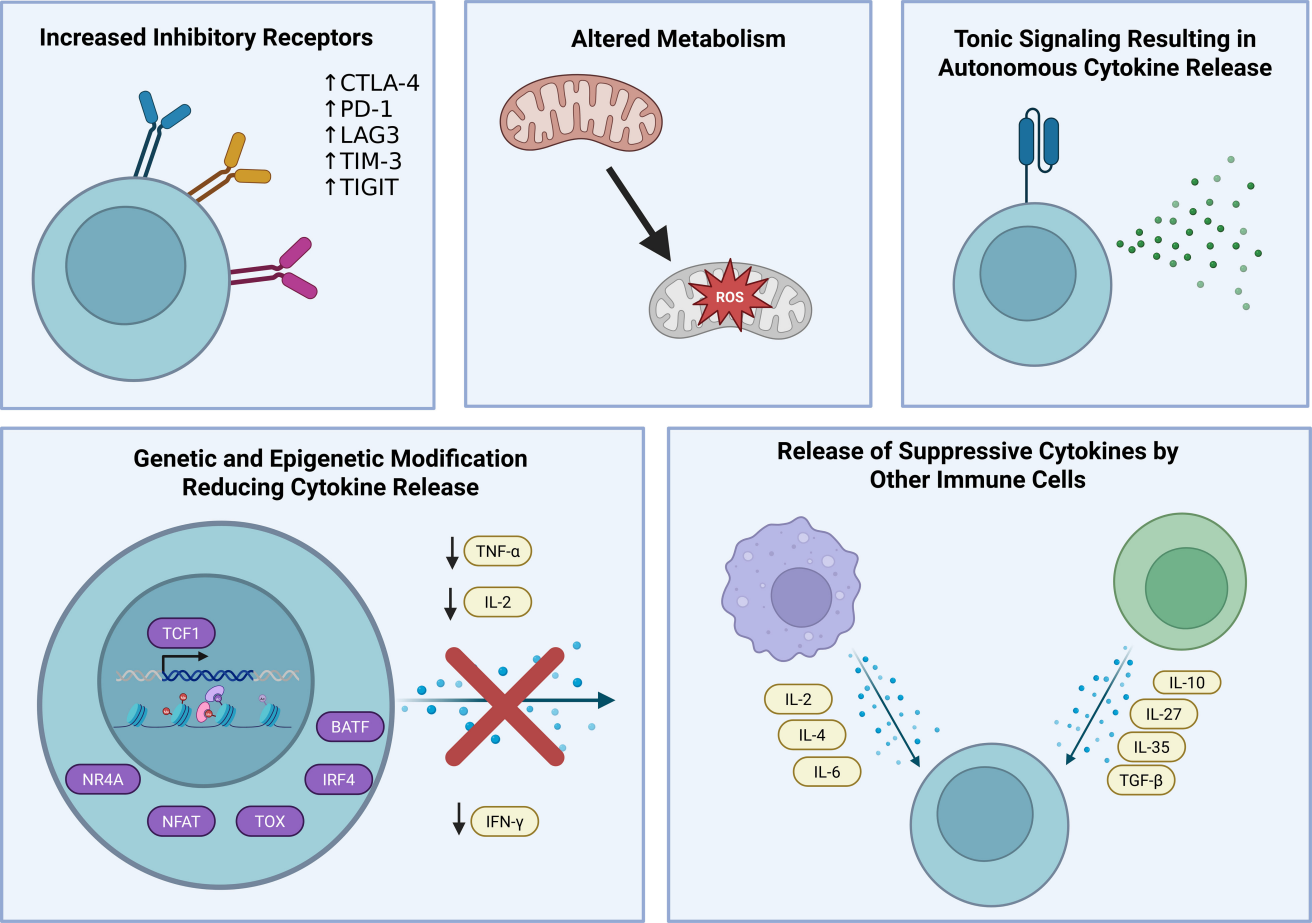
T cell exhaustion is characterized by phenotypic changes including increased expression of inhibitory receptors, altered metabolism and aberrant cytokine release from recurrent antigen exposure and tonic signaling, genetic and epigenetic reprogramming, and altered functionality from release of suppressive cytokines by other immune cells. Figure created in https://BioRender.com.

Native T-cell exhaustion evolved as a mechanism to limit damage to host tissues during periods of persistent inflammation and can broadly be defined as the loss of T-cell effector and regenerative functions resulting from prolonged antigen exposure (**Figure 1**) (32). Although physiological, exhaustion of T-cells leads to an attenuated immune response, which may reduce the control of chronic infections and cancer and allow for disease persistence.

With initial antigen exposure, naïve T-cells expand rapidly and acquire effector functions that mediate cytotoxicity, including cytokine production. Most of these T-cells will undergo apoptosis, and a minor population further differentiates into memory T-cells (33). However, in the setting of chronic antigen exposure such as with chronic viral infections, antigen-specific T-cells are unable to differentiate appropriately into memory T-cells and instead undergo progressive functional decline and depletion over time (34, 35). These exhausted T-cells are characterized by increased expression of multiple inhibitory mediators, including PD1, TIM3, LAG3, CTLA4, and TIGIT, and characteristically exhibit functional attenuation in response to TCR activation. Several key transcription pathways, involving transcription factors TCF-1, TOX, NFAT, IRF4, BATF and NR4A, have been shown to drive genetic and epigenetic alterations mediating attenuation of effector functions in exhausted T-cells (36). These cells exhibit loss of cytokine production, starting with IL-2, followed by TNF-α and IFN-γ which summarily impairs effector and proliferative function (37). Furthermore, chronic antigen stimulation induces transcriptional repressors such as Blimp-1, which impair mitochondrial biogenesis and function, thus leading to increased formation of reactive oxygen species (ROS) that drive exhaustion by inhibiting phosphatases and sustaining aberrant NFAT signaling (38). Although a subset of exhausted T-cells may be re-activated and even proliferate in response to antigen, repeated antigen stimulation ultimately leads to a state of terminal exhaustion, characterized by a loss of transcription factor TCF1 (39). At this stage, further T-cell proliferation and effector activity is permanently arrested (40, 41).

Tumor antigens are often weakly immunogenic due to sequence similarity to self-antigens. Consequently, T-cells targeting these antigens arise in lower frequencies and bind with lower affinity, thereby reducing their overall effectiveness (41). Even after effector T-cells infiltrate the tumor microenvironment, their function can be suppressed by the activities of multiple tumor associated immunosuppressive cells, including tumor-associated macrophages, mature regulatory dendritic cells, cancer-associated fibroblasts, myeloid derived suppressor cells, and regulatory T-cells. These populations trigger exhaustion both through direct cell-cell interaction and by secretion of suppressive cytokines, such as TGF-β, IL-4, IL-6, IL-10, IL-27, and IL-35 (32, 42–47). Furthermore, the tumor microenvironment is hypoxic and nutrient poor, creating hostile conditions for T-cells by impairing mitochondrial function, increasing ROS, and impairing metabolic signaling (32, 38, 48, 49). Together, these immunologic, metabolic, and structural barriers converge to reprogram infiltrating T-cells toward a dysfunctional, exhausted phenotype, ultimately limiting effective anti-tumor activity.

## CAR-T cell exhaustion

**Figure 2:**
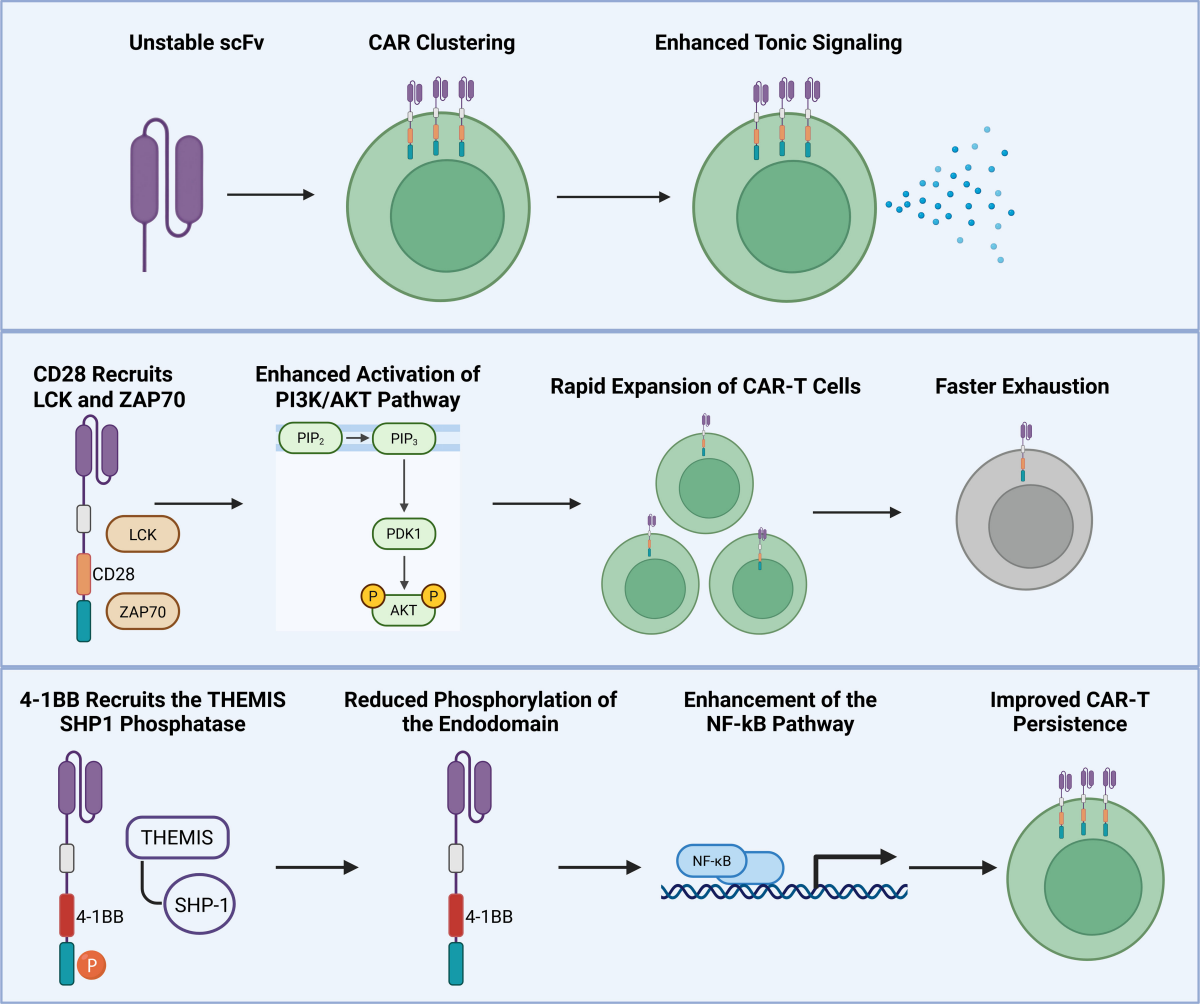
CAR-T cell exhaustion is similar in many ways to native T cell exhaustion but also exhibits unique exhaustion pathways due to activity of individual components of the CAR construct, including the scFv and costimulatory domains. Figure created in https://BioRender.com.

Most chimeric antigen receptor constructs are composed of several functional domains: an antigen binding domain (typically a single-chain variable fragment, scFv), a hinge region, a transmembrane domain, and an endodomain composed of both a co-stimulatory (typically 4-1BB or CD28) and CD3ζ signaling domain (49). Upon engagement with their cognate antigen, the CAR initiates intracellular signaling cascades that resemble canonical TCR-mediated activation, including induction of transcriptional, metabolic, and functional programs necessary for T-cell effector function (50). Thus, CAR-T cells are also susceptible to developing an exhausted phenotype in a manner analogous to exhaustion in native T-cells (**Figure 2**). The timing and kinetics of CAR-T cell exhaustion *in vivo* remain poorly elucidated; however, an *in vitro* tumor culture model has demonstrated the emergence of exhaustion markers within seven days of sustained antigen exposure (51). Although tonic antigen stimulation through the CAR likely contributes to CAR-T cell exhaustion via similar mechanisms as the TCR, unique mechanisms specific to CAR-T cell exhaustion have also been identified.

### Tonic signaling

In a process termed “tonic signaling,” native T-cells routinely engage with MHC-presented self-peptide antigens, thereby maintaining a low-level of constitutive activity necessary for maintaining functional readiness and survival (52). An analogous process has been observed to occur intrinsically through CAR constructs, with low-level basal signaling occurring independently of target antigen engagement (53). Although the specific mechanisms of tonic signaling in CARs have not been fully elucidated, it has been demonstrated that the instability of the scFv can promote self-aggregation, thereby triggering downstream signaling through the endodomain in the absence of target antigen (54) (**Figure 2**). Additionally, positively charged amino acids within the antigen binding domain, termed positively charged patches (PCP’s), have been associated with enhanced CAR clustering and tonic activation (53). This heightened tonic signaling results in improved immunologic synapse formation as well as activation of pro-inflammatory gene expression, potentially augmenting effector activity (53). Conversely, this excess tonic signaling may paradoxically impair CAR-T cell efficacy by accelerating the onset of exhaustion (31, 52, 53). Moreover, in patients with B-cell malignancies, CD8+ CAR-T cells isolated from non-responders demonstrate transcriptional programs consistent with terminal exhaustion, including increased expression of TOX and EOMES, suggesting a more terminally differentiated dysfunctional state that may be less amenable to reinvigoration strategies (55, 56).

### Co-stimulatory domain

Most CAR constructs utilize either a CD28 or 4-1BB co-stimulatory domain, and each modulates CAR-T cell function through distinct signaling pathways. CD28 is a co-stimulatory receptor that is constitutively expressed on both resting and activated native T-cells. Upon binding to its cognate ligands, CD80 or CD86, CD28 induces a signaling cascade that activates several transcription factors, including NFAT, AP-1 and NF-κB, to promote IL-2 expression, T-cell proliferation, and survival (57). Additionally, CD28 amplifies tonic signaling by recruiting kinases including LCK and Zap70 which promote downstream activity in the PI3K/AKT pathway (58) (**Figure 2**). This amplified signaling results in increased early CAR-T cell activation and expansion *in vitro*; however, this has also been shown to correlate with a higher degree of exhaustion and inferior persistence compared to 4-1BB co-stimulated CAR-T cells *in vivo* (31, 59).

4-1BB, unlike CD28, is not expressed on resting T-cells and is only upregulated upon T-cell activation. Activation of 4-1BB recruits TNFR-associated factors (TRAFs), which subsequently leads to activation of NF-κB and proliferation of T-cells (57). In contrast to CD28, 4-1BB co-stimulated CAR constructs mitigate the effects of tonic signaling by reducing the phosphorylation of the endodomain through recruitment of the THEMIS-SHP1 phosphatase complex, by activating the NF-kB pathway to enhance T-cell survival, and by reducing activation of pro-apoptotic pathways, resulting in less exhaustion and superior persistence (31, 60–62) (**Figure 2**).

Importantly, neither preclinical nor clinical studies have suggested a clear consensus on the superior CAR co-stimulatory domain for anti-tumor activity. While augmented tonic signaling is associated with the CD28 co-stimulatory domain, it is also associated with higher cytokine release which can promote its effector function but also increase off-target toxicity (57). The 4-1BB co-stimulatory domain is associated with reduced tonic signaling and higher persistence, though this also correlates with slower tumor clearance than with CD28 (57). Currently, there are no direct randomized clinical trials comparing the efficacy of CAR-T constructs containing CD28 and 4-1BB and only retrospective comparisons have been made between trials evaluating CAR-T cell products bearing each of these co-stimulatory domains (63–71) (**Table 2**).

**Table 2:** Studies comparing axi-cel (CD28) vs liso-cel (4-1BB).

Author (Year)	Population Investigated	Number of Patients	Lymphoma Type	Efficacy Outcomes (Axi-cel vs Liso-cel)	Conclusions	Safety outcomes
Matched-Adjusted Indirect Comparison						
Maloney et al (2021) (63)	TRANSCEND & ZUMA-1	256 (liso-cel) 101 (axi-cel)	DLBCL (3L+)	ORR; CR; PFS; OS	No significant difference in efficacy outcomes	Axi-cel had higher rate of CRS & ICANS
Oluwole et al (2022) (64)	TRANSCEND & ZUMA-1	215 (liso-cel) 51.5 (axi-cel)	DLBCL (3L+)	ORR; CR; PFS; OS; DOR	Axi-cel had higher PFS & OS	Axi-cel had higher rate of CRS & ICANS
Abramson et al (2024) (65)	TRANSFORM & ZUMA-7	80 (liso-cel) 359 (axi-cel)	DLBCL (2L)	ORR; CR; EFS; PFS	No significant difference in efficacy outcomes	Axi-cel had higher rate of CRS & ICANS
Boardman et al (2025) (66)	TRANSCEND FL & ZUMA-5	101 (liso-cel) 124 (axi-cel)	FL (3L+)	ORR; CR; PFS; OS; DOR; TTNT	Liso-cel had higher CR rate	Axi-cel had higher rate of CRS & ICANS
Retrospective and Meta-analyses						
Meng et al (2021) (67)	Meta-analysis	2,172 (including tisa-cel)	Multiple	ORR; CR	No significant difference in efficacy outcomes	Axi-cel had higher rate of CRS & ICANS
Tun et al (2023) (68)	Retrospective study of 7 academic centers	49 (liso-cel) 131 (axi-cel)	DLBCL (2L or 3L+)	ORR; CR; PFS; OS	No significant difference in efficacy outcomes	Not compared
Ali et al (2024) (72)	Retrospective analysis of CIBMTR data	5,256 (including tisa-cel)	DLBCL (all lines)	ORR	No significant difference in efficacy outcomes	Axi-cel had higher rate of CRS & ICANS
Portuguese et al (2025) (69)	Retrospective study of a single center (Fred Hutchinson Cancer Center)	160	DLBCL (all lines)	ORR; CR; PFS; OS; DOR	No significant difference in efficacy outcomes	Axi-cel had higher rate of CRS & ICANS
Looka et al (2025) (70)	Retrospective study or a single center (Dana Farber Cancer Center)	37 (liso-cel) 50 (axi-cel)	DLBCL (3L)	ORR; PFS; OS; DOR	Axi-cel had higher ORR & PFS	Axi-cel had higher rate of CRS & ICANS
Yu et al (2024) (71)	Retrospective study of TriNetX data	179 per cohort*	DLBCL (all lines)	CR; PFS; OS	No significant difference in outcome	Axi-cel had higher rate of ICANS

*MAIC, Matched-Adjusted Indirect Comparison; 2L, second line treatment for lymphoma; 3L+, third line (or later) treatment for lymphoma; ORR, objective response rate; CR, complete response; PFS, progression free survival; OS, overall survival; EFS, event free survival; DOR, duration of response; CRS, cytokine release syndrome; ICANS, immune effector cell-associated neurotoxicity-.

### Inhibitory receptors and transcriptional programs of CAR-T exhaustion

Exhaustion associated inhibitory receptors, including CTLA-4, PD-1, LAG-3, TIM-3, and TIGIT, have been implicated in CAR-T cell dysfunction, paralleling the effects seen in native T-cell exhaustion. The expression of these canonical exhaustion markers is directly regulated by interconnected transcriptional pathways. The high mobility group (HMG)-box transcription factors of the TOX family are central mediators of this program, functioning in cooperation with NR4A family transcription factors in a positive feedback loop to drive expression of multiple inhibitory receptors. In CAR-T cells, TOX and TOX2 are markedly induced in CD8+ CAR PD-1+ TIM-3+ exhausted tumor-infiltrating lymphocytes, and dual knockout has been shown to reduce inhibitory receptor expression while increasing cytokine production and improving tumor control (73).

BATF is another key transcription factor which appears to have context and dose dependent effects on directing CAR-T cell fate. In states of chronic antigen stimulation, elevated BATF activity leads to upregulation of exhaustion related transcriptional programs, including those encoding PD-1. In preclinical CAR-T models, it was demonstrated that knockout of BATF improves resistance to exhaustion and shifts populations toward a central memory phenotype (51, 74).

Pre-treatment exhausted or senescent phenotypes within manufactured CAR-T products have also been associated with poor clinical outcomes (55). However, inhibitory receptor expression does not universally indicate irreversible dysfunction. In some cohorts, increased PD-1 and LAG-3 expression specifically on CD4+ CAR-T cells at peak expansion, combined with reduced expression of CD107a (a marker of cytotoxic activity) paradoxically may predict improved long-term disease control (75). Collectively, these findings suggest that early upregulation in exhaustion-associated markers during the acute phases of activation and expansion may reflect physiologic activation rather than true exhaustion, whereas sustained inhibitory receptor expression in conjunction with impaired effector functions may be more consistent with a truly exhausted CAR-T cell phenotype. Further studies are needed to better define and distinguish these functional states in clinical CAR-T therapy.

### Extrinsic drivers of CAR-T cell exhaustion

The tumor microenvironment (TME) also impacts CAR-T cell anti-tumor response through the presence of immunoregulatory cell populations, altered metabolic processes and through inhibitory cell-cell interactions.

Immunosuppressive cells that infiltrate the TME, including Myeloid-derived suppressor cells (MDSCs), regulatory T-cells (Tregs), and tumor-associated macrophages (TAMs), mediate CAR-T cell exhaustion and often vastly outnumber CAR-T cells in the TME. *Post-hoc* analysis from the ZUMA-1 trial, which investigated axicabtagene ciloleucel in patients with relapsed/refractory large cell lymphoma, demonstrated that patients with high baseline monocytic MDSC levels (>250 cells/μL) had a four-fold lower peak CAR-T cell expansion than those with lower baseline levels (76). Notably, CAR-T cell activity itself may further remodel the local immune environment, promoting inflammation that triggers expansion of MDSCs and other immunosuppressive cell populations, potentially mediated by interferon responses, hypoxia-associated pathways and TGF-β signaling (77).

In contrast, a TME characterized by higher expression of certain chemokines (e.g., CCL5 and CCL22), cytokines (e.g., IL-15, IL-7, IL-21) and interferon-regulated molecules has been associated with enhanced CAR-T cell trafficking, CAR-T activity signatures, and improved clinical outcomes (78). Among patients with high tumor burden treated with axicabtagene ciloleucel, a favorable microenvironment with reduced suppressive myeloid cell activity (low *ARG2* and *TREM2* expression) has also been associated with a more durable response (79).

Metabolic stress within the tumor microenvironment also contributes to exhaustion through nutrient depletion, hypoxia, and accumulation of immunosuppressive metabolites. CAR-T cells exposed to a hypoxic and nutrient poor TME may exhibit metabolic reprogramming from oxidative phosphorylation toward an increased reliance on glycolysis, a shift that has been associated with reduced self-renewing capacity and anti-tumor activity (80). Hypoxia-driven signaling pathways, including HIF-1α and its downstream target VEGF, as well as TGF-β, further promote T-cell dysfunction through multiple interconnected mechanisms (77). Furthermore, elevated mitochondrial reactive oxygen species (ROS) generated during chronic antigen exposure have been shown to contribute to exhaustion through oxidative stress-induced telomere fragility, activating DNA damage response pathways that restrict proliferation and impair cytokine production (81). Lactic acid accumulation resulting from glycolysis also creates an acidic microenvironment that directly contributes to dysfunction, which may be driven by terminally exhausted T cells uniquely upregulating the monocarboxylate transporter 11 (MCT11) (82). Cholesterol metabolism also appears to play a critical role in driving exhaustion, with C1QB+ M2 macrophages driving an inflammatory state through cholesterol efflux, leading to reduced CAR-T cell cytotoxicity (83).

Cell-cell interactions also play a key role in modifying the tumor microenvironment and affecting CAR-T cell efficacy. Uncovering the tumor architecture through single cell spatial analysis revealed cellular neighborhoods containing more immune cells are associated with longer remission, while fibroblasts and vascular endothelial cells residing closer to tumor cells correlate with poor response (84). Furthermore, CSF1R+ CD14+ CD68+ lymphoma-associated myeloid-monocytic (LAMM) cells infiltrate the tumor microenvironment to impair CAR T-cell function through direct cell-cell interaction via the PGE2-EP2/EP4 axis (85).

## Strategies to combat CAR-T cell exhaustion

### Strategies to optimize the CAR construct

Emerging evidence suggests that exhaustion can be mitigated through rational engineering of the antigen-binding domain, signaling elements, and regulatory features of the CAR construct.

### Optimization of the antigen binding domain

As discussed previously, the CAR antigen binding domain (scFv) engages in tonic signaling through aggregation and auto-activation of downstream functions through the CD3ζ signaling domain (54). This phenomenon is driven by intrinsic instability within the scFv, which is dependent on specific amino acid residues within the framework regions of the variable domains. Modeling with computational mutagenesis has identified key amino acid changes that are most likely to cause this instability, specifically by identifying mutations that conferred greater thermodynamic instability within the scFv. By swapping out these amino acids for those that increase the stability of the scFv, signaling through the CAR was mitigated without compromising antigen specificity (54). Humanization of murine derived scFv framework regions also reduced tonic signaling and led to improved anti-tumor effects of these CAR-T cells *in vitro* (54).

The affinity of the scFv also impacts the efficacy and off-target effects of CAR-T cell therapy. Although increasing the target affinity of tumor-specific TCRs has been shown to promote receptor clustering, cellular signaling, proliferation, and target cell lysis, preclinical evidence suggests that there is a threshold above which increased affinity is no longer optimal for cytotoxic T-cell function (86). Most CAR T constructs in clinical practice today utilize high-affinity scFvs, which may drive excessive activation, thus leading to reduced persistence and increased off-target toxicity (87). To address these concerns, low affinity CARs have been designed to have a higher dissociation equilibrium constant (*K_D_)* than that of traditional CARs. In preclinical models, low-affinity CAR-T cells exhibited significantly greater antigen specific proliferation, *in vivo* anti-tumor activity, and increased expression of CD127 (IL-7Rα) and B-cell lymphoma-2 (BCL-2), leading to improved survival and persistence (88). Interestingly, no differences were observed in expression of exhaustion markers between low affinity and control CAR T cells (88).

Clinically, a low-affinity, autologous 4-1BB anti-CD19 CAR-T cell product has been evaluated in pediatric patients with relapsed/refractory B-cell acute lymphoblastic leukemia (B-ALL) and showed strong 3-month molecular remission (86%) and 12-month overall survival (63%), with a median duration of CAR-T cell persistence of 215 days (88). Another low-affinity CAR-T cell product, obecabtagene autoleucel (obe-cel), has recently been approved by the US Food and Drug Administration for the treatment of adults with relapsed or refractory B-ALL. In the pivotal phase 1–2 FELIX trial, obe-cel demonstrated a 57% complete remission (CR) rate, with a median duration of response of 14.1 months (89). Notably, the median duration of CAR-T cell persistence in this study was 17.8 months (89). Similarly designed low affinity anti-CD19 CAR-T cell products have not yet been investigated clinically in non-Hodgkin’s Lymphoma (NHL). However, preclinical development of three novel CARs targeting CD79B for treatment of NHL has shown that the product associated with the lowest relative antigen affinity was associated with reduced antigen loss of CD79b and the highest *in vivo* efficacy (90).

### Optimization of the co-stimulatory domain

As previously discussed, CD28 and 4-1BB co-stimulatory domains have different effects on tonic CAR signaling, with CD28-based CARs demonstrating a greater propensity for basal activation. It was therefore hypothesized that targeted mutagenesis within the CAR construct could reduce tonic activation and subsequent expression of exhaustion-associated genes. This strategy initially demonstrated success when replacing amino acids within the CD3ζ signaling domain resulted in less CAR activation and promoted differentiation of the cell into a memory-like phenotype (91). Building on this approach, it was demonstrated that by introducing null mutations to the CD28 subdomain, T-cells expressing these mutated CARs had increased sensitivity to antigen stimulation, higher cytokine production after repeated antigen and PD-L1 exposure, and reduced expression of exhaustion-associated transcription factors (92). These findings highlight promising mechanisms to limit CAR-T cell exhaustion through mutagenic alterations of the CAR construct. Recently, a novel CD19 CAR-T product with a modified CD28 costimulatory domain, 1XX, was developed to calibrate signaling by introducing inactivating point mutations in the two distal immunoreceptor tyrosine-based activation motifs. In a first-in-human phase I trial, treatment with CD19-1XX CAR-T cells in 28 patients with relapsed/refractory LBCL resulted in an overall response rate of 82% and a CR rate of 71%. Additionally, 14 of 16 patients (~88%) treated at the dose level chosen for expansion remained in CR after 12 months. The 1XX CAR-T products notably also contained a higher proportion of memory CD8 T cells and CAR-T cell persistence was detected beyond 1–2 years in patients with ongoing remission (93).

Recently, third generation CARs have been developed to incorporate dual co-stimulatory domains within a single construct. This combined co-stimulatory approach allows for incorporation of very low affinity antigen binding domains, which may allow for reduced CAR-T cell exhaustion with augmented efficacy and safety (94). Preclinical studies have shown that these CARs can promote enhanced proliferation, persistence, and cytotoxicity, with the potential for improved durability of anti-tumor responses (95, 96). In a prior phase I study, 16 patients with relapsed/refractory B cell NHL were simultaneously infused with a second-generation CAR containing a CD28 costimulatory domain and a third generation CAR containing both costimulatory domains. Expansion of the third generation CAR was notably higher two weeks after infusion and increased molecular signaling from the third generation CAR was noted 6 months after infusion in surviving patients (97). Similarly, integrating this concept is the novel bicistronic CAR-T cells incorporating dual targeting with both anti-CD19 CAR with a CD28 co-stimulatory domain and anti-CD20 CAR with a 4-1BB co-stimulatory domain, which promises to overcome current CAR-T cell therapy limitations due to tumor heterogeneity (98). Early clinical trials demonstrated pronounced CAR-T expansion, accompanied by favorable anti-lymphoma and safety profiles (99).

### Fourth and fifth generation CARs

Fourth generation “armored” CAR-T cells, also called *T cells redirected for universal cytokine-mediated killing* (TRUCKs) have also been engineered to secrete immune-stimulatory cytokines such as interleukin-18 (IL-18), which enhance both the intrinsic activity of the CAR-T cell and the broader anti-tumor immune response (100). Notably, IL-18-secreting CAR-T cells have demonstrated clinical activity in patients with relapsed/refractory lymphoma who previously failed treatment with a second-generation anti-CD19 CAR-T cell therapy. In a recently published phase 1 trial, 11 of 21 patients who received the fourth generation armored CAR-T product, huCART-IL-18 achieved a CR at 3 months and the median duration of response was 9.6 months (101).

**Figure 3:**
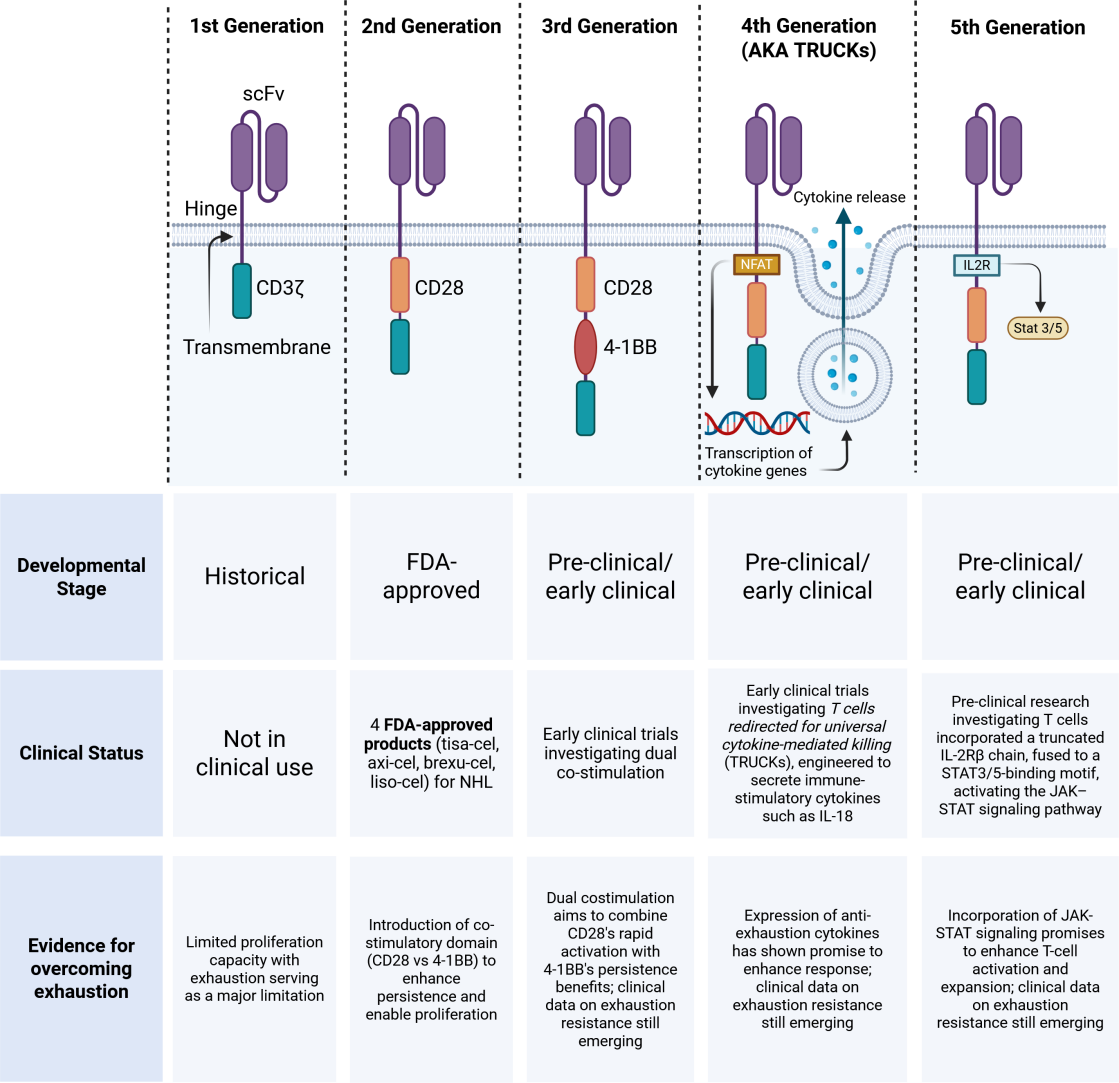
Generations of chimeric antigen receptors (CAR) and current clinical status second-generation CARs with costimulatory domains (CD28 or 4-1BB) are FDA-approved for hematologic malignancies. Newer generation CARs are under investigation to enhance persistence and overcome effects of exhaustion. Figure created in https://BioRender.com.

More recently, fifth-generation CAR-T cells capable of induced cytokine signaling have been investigated as an innovative platform to improve CAR-T cell efficacy and avoid exhaustion. The prototypical fifth generation CAR-T cells incorporated a truncated interleukin-2 receptor β (IL-2Rβ) chain, fused to a STAT3/5-binding motif (102). Upon antigen engagement, this domain activates the JAK–STAT signaling pathway, providing a third signal that mimics physiological cytokine stimulation and enhances T-cell activation, expansion, and persistence in an antigen-dependent manner (103). Another fifth-generation CAR-T cell product, designed with three co-stimulatory domains and the ability to secrete anti-PD-L1 scFv has been demonstrated to exhibit enhanced cytotoxicity and reduced exhaustion in *in vitro* CD19 and BCMA expressing tumor cell models (104, 105). These advanced designs may represent the next leap forward in optimizing CAR-T cell potency, resistance to exhaustion, and long-term efficacy (**Figure 3**).

### Gene editing strategies to enhance CAR-T cell function

The emergence of CRISPR/Cas9 technologies has enabled a unique capability to perform targeted gene knockout and safe and efficient gene insertion to modify CAR-T cells. Traditionally, CAR constructs have been transduced into T-cells through retroviral or lentiviral vectors, resulting in untargeted genomic integration. In contrast, the emergence of CRISPR/Cas9-based genomic targeting has promised more efficient and targeted gene integration. Preclinical research has demonstrated that inserting the CAR in the TRAC locus enhances tumor killing, while delaying T-cell differentiation and exhaustion (106). Furthermore, CRISPR based knockout of checkpoint inhibitors, PD-1 and CLTA-4, the inhibitory receptor TGF-β II, and negative modulator Cbl-b show preclinical promise in reversing exhaustion and enhancing T-cell function (107–113). Early clinical experience has supported the feasibility of this approach. A phase I study investigating a CRISPR/CAS9 edited CAR-T product incorporating the CAR sequence into a PD1 locus has demonstrated strong clinical activity in patients with relapsed/refractory NHL, with a CR rate of 86% and a median PFS exceeding 19 months (114).

Other methods studied to mitigate PD-1 signaling include modification of the CAR-T cell to express a dominant negative PD-1 receptor, which blocks inhibitory signaling after PD-1/PD-L1 interaction. In a phase 1 trial including 9 patients with relapsed/refractory NHL who were treated with dominant-negative PD-1 armored anti-CD19 CAR-T cells, the ORR was 78% and the CR rate was 55.6% (115). CRISPR-based screening strategies are also being adapted to identify novel markers of exhaustion that can be targeted in future studies (116).

Moreover, increased expression of proteins that reduce susceptibility to apoptosis is also being investigated to extend the lifespan of CAR-T cells by reducing activation-induced cell death (AICD). In a preclinical study in which the anti-apoptotic proteins Bcl-2 and Bcl-xL were overexpressed in investigational CAR-T cells, there was an improvement in both expansion and persistence. Interestingly, the CAR-T cells overexpressing BCL-xL also demonstrated reduced exhaustion and thus had enhanced cytotoxic function *in vivo* compared to control CAR-T cells (117). Based on these findings, an armored CAR-T cell product modified to express BCL-xL was tested in murine models and demonstrated enhanced efficacy and resistance to exhaustion (118).

### Targeting TOX and TGF-β

Thymocyte selection-associated HMG BOX (TOX) is a transcription factor that is expressed during the normal development of helper T and NK cells. In activated T-cells, TOX has been identified as a major transcriptional mediator of T-cell exhaustion (119). An increase in TOX expression has been directly associated with the development of an exhausted T-cell phenotype through the enhanced transcription of multiple exhaustion related genes (120, 121). In murine studies, knockout of TOX, or a related transcription factor, NR4A, in CAR-T cells led to enhanced anti-cancer activity and promoted T-cell survival (122).

Transforming growth factor-β (TGF-β), an immunosuppressive cytokine abundantly expressed in many tumor microenvironments, has also been shown to promote T-cell exhaustion and dysfunction via multiple pathways (123). To counteract this, CAR-T cells have been engineered to target and neutralize TGF-β, either by removing soluble TGF-β from the microenvironment or by modifying cell signaling mechanisms to produce immunostimulatory responses upon TGF-β binding (108, 124). TGF-β–resistant CAR-T cells represent a promising strategy in the design of next-generation CAR-T cell therapies to overcome immunosuppression and enhance therapeutic durability.

## Adjunctive therapeutic strategies

Numerous adjunctive therapeutic strategies are being explored to overcome CAR-T cell exhaustion and improve clinical outcomes. These approaches target distinct biological pathways related to T-cell exhaustion and aim to restore or preserve CAR-T cell fitness through combinatorial or sequential interventions (**Tables 3**, **4**) (101, 125–133).

**Table 3:** Prior CAR-T combination trials.

Study (Year)	Intervention	Results
Sequential Anti-CD19 Directed Chimeric Antigen Receptor Modified T-Cell Therapy (CART19) and PD-1 Blockade with Pembrolizumab in Patients with Relapsed or Refractory B-Cell Non-Hodgkin Lymphomas (2018) (125)	After relapse of NHL treated with CAR-T, pembrolizumab 200mg was administered every 3 weeks until progression of disease or unacceptable toxicity	Of 12 patients, best ORR was 27% [1 CR, 2 PR, 1 had stable disease, and 7 had progressive disease]
Phase 1/2 primary analysis of ZUMA-6: Axicabtagene ciloleucel (Axi-Cel) in combination with atezolizumab (Atezo) for the treatment of patients (Pts) with refractory diffuse large B cell lymphoma (DLBCL) (2020) (126)	In Phase 1, atezolizumab was given at 1200 mg every 21 days for 4 doses starting on Day 21, 14, and 1 post-axi-cel infusion for Cohorts 1, 2, and 3, respectively. Patients received the Cohort 3 atezolizumab (Day +1) dosing schedule in Phase 2.	Of 28 patients, best ORR was 75%. Efficacy outcomes were overall similar to those treated with atezolizumab compared with those treated with axi-cel alone
JCAR014 and Durvalumab in Treating Patients With Relapsed or Refractory B-cell Non-Hodgkin Lymphoma (2022) (127)	Group 1: Patients received JCAR014 on day 0 and durvalumab on day 21 and then every 4 weeks for up to 10 doses Group 2: Patients received durvalumab on day –1, JCAR014 on day 0, then up to 10 additional doses every 4 weeks in the absence of disease progression or unacceptable toxicity	Study terminated due to slow accrual
Anti-CD19 CAR T cells in combination with ibrutinib for the treatment of chronic lymphocytic leukemia (2022, 128)	Patients with CLL who had not achieved better than PR after ≥6 months of ibrutinib were treated with autologous anti-CD19 humanized binding domain T cells	Of 20 patients, CR rate was 43% at 3 months
Safety and efficacy of tisagenlecleucel plus pembrolizumab in patients with r/r DLBCL: phase 1b PORTIA study results (2023) (129)	Pembrolizumab 200 mg was administered every 3 weeks on D-1, D8, or D15 for up to 6 doses until progression of disease or unacceptable toxicity	Of 12 patients, best ORR was 50%. 4 (33.3%) had CR, 2 (16.7%) had PR, and 6 (50%) had PD
A Safety and Efficacy Trial of JCAR017 Combinations in Subjects with Relapsed/Refractory B-Cell Malignancies (2024) (130)	JCAR017 given in combination with durvalumab, CC-122 (a pleiotropic pathway modifier), iberdomide, ibrutinib, retalimab and/or nivolumab, or golcadomide	Completed accrual
CAR T cells and time-limited ibrutinib as treatment for relapsed/refractory mantle cell lymphoma: the phase 2 TARMAC study (2024) (131)	Ibrutinib was given for a minimum of 7 days before leukapheresis and continued throughout CAR-T manufacturing, lymphodepletion, and after CAR-T infusion. For those in complete response (CR) and without MRD by flow cytometry at 10–^5^ in peripheral blood at 6 months after infusion, ibrutinib was ceased	Of 20 patients, 80% of patients demonstrated CR. At 13-month median follow-up, the estimated 12-month PFS was 75% and OS was 100%
BTK Inhibitor Synergizes with CD19-Targeted Chimeric Antigen Receptor-T Cells in Patients with Relapsed or Refractory B-Cell Lymphoma: An Open-Label Pragmatic Clinical Trial (2024) (132)	Treatment with CAR-T monotherapy or in combination with ibrutinib, zanubrutinib, or orelabrutinib	Of 37 patients, 13 received combination therapy with a BTKi. Best ORR were 84.6% vs 66.7% and best CR were 61.5% vs 25.0% in patients with and without BTKi
Cluster of Differentiation Antigen 19/22 CAR T Cells (AUTO3) for the Treatment of Diffuse Large B Cell Lymphoma (ALEXANDER) (2025) (133)	Limited duration pembrolizumab during pre-conditioning or consolidation given in combination with CD19/22 CAR-T AUTO3	Study terminated after review of preliminary data from phase 1 portion
Enhanced CAR T-Cell Therapy for Lymphoma after Previous Failure (2025) (101)	Interleukin-18 secreting armored CAR-T cell administered to patients after failure of prior treatment with CD19 CAR-T	Of 21 patients, the ORR was 81%, CR was 52%, and PR was 29% at 3 months

**Table 4:** Currently active or recruiting CAR-T combination trials.

National clinical trial identifier	CAR-T product and combination agent	Disease subtype
NCT05659628	CAR-T expressing IL-7 and CCL19 and tislelizumab	R/R DLBCL
NCT05052528	CD19 CAR-T and rituximab beforehand	R/R DLBCL
NCT05495464	Brexucabtagene autoleucel with rituximab and acalabrutinib	Newly diagnosed high-risk mantle cell lymphoma
NCT04889716	CD19 CAR-T followed by mosunetuzumab or obinutuzumab and glofitamab	R/R DLBCL or transformed FL
NCT05260957	CD19 CAR-T followed by mosunetuzumab and polatuzumab	R/R aggressive NHL
NCT05633615	Tisagenlecleucel, axicabtagene ciloleucel, or lisocabtagenee maraleucel followed by mosunetuzumab and polatuzumab	R/R DLBCL
NCT03960840	Rapcabtagene autoleucel and ibrutinib	CLL with stable disease or partial response after 6 months of second or subsequent line ibrutinib
NCT05672173	Lisocabtagene maraleucel, nivolumab, and ibrutinib	Richter’s transformation
NCT05744037	CD19 CAR-T and zanubrutinib	R/R B cell lymphoma
NCT05202782	CD19 CAR-T and zanubrutinib	R/R aggressive B-cell NHL or transformed indolent B-cell lymphoma
NCT05873712	Lisocabtagene maraleucel and zanubrutinib	Richter’s transformation
NCT04257578	CD19 CAR-T and acalabrutinib	B cell lymphoma
NCT04484012	CD19 CAR-T and acalabrutinib	R/R mantle cell lymphoma
NCT05359211	Lisocabtagene maraleucel and IL-15 receptor agonist NKTR-255	R/R LBCL
NCT05432635	CMV-Specific CD19 CAR-T following stem cell transplantation and CMV vaccine	B cell NHL
NCT05801913	CMV-Specific CD19 CAR-T and CMV vaccine	B cell NHL

### Checkpoint inhibition

As previously noted, persistent antigen exposure leads to increased expression of inhibitory immune checkpoint receptors, including PD-1, TIM-3, LAG-3, TIGIT and CTLA-4 on T-cells, characterizing the exhausted phenotype (37). Furthermore, high expression of programmed cell death–ligand 1 (PD-L1) on pre-treatment lymphoma cells has been associated with inferior outcomes to CAR-T therapy, and addition of checkpoint inhibitor (CPI) therapy has been shown to reduce exhaustion and improve anti-tumor efficacy in preclinical models (134, 135). Thus, combining CPI monoclonal antibodies with CAR-T therapy has emerged as a promising strategy for combating CAR-T cell exhaustion.

Despite the theoretical benefit of combination CPI-CAR-T cell therapy, the results of several early phase clinical trials have failed to indicate substantive signals of improved clinical outcomes with CPI-CAR-T combinations (126, 129, 136, 137). For example, in the ZUMA-6 study 28 patients with relapsed or refractory diffuse large B-cell lymphoma (DLBCL) received axi-cel with the PD-L1 blocking antibody, atezolizumab (126). The toxicity, efficacy, and CAR-T cell expansion appeared to mirror those of axi-cel monotherapy. In another study, patients with LBCL treated with another PD-L1 blocking antibody, durvalumab, prior to CAR-T therapy demonstrated inferior efficacy, which was associated with delayed CAR T expansion, increased soluble PD-L1 and lower concentrations of inflammatory cytokines in the blood (136). Despite this, ongoing durvalumab therapy after CAR-T infusion was associated with later CAR-T re-expansion and prolonged response duration, suggesting that the timing of CPI therapy may be important for this therapeutic combination.

Preclinical evidence also suggests that CTLA-4 disruption may be particularly effective in enhancing CAR-T cell function, as CTLA-4 deficiency permits unopposed CD28 co-stimulatory signaling and sustained CAR expression; however, clinical activity supporting CTLA-4 disruption in the context of CAR-T therapy is limited (111). Another preclinical study has shown a possible synergistic antitumor effect of PD-1 and TIGIT downregulation on CAR-T cells, with downregulation of TIGIT specifically resulting in less CAR-T cell exhaustion. Currently, CRC01, a CD19 CAR-T product with PD-1 and TIGIT genes silenced by shRNAs, is being investigated in a phase I/II study in patients with relapsed/refractory LBCL (138).

More recently, a larger study investigated the effect of CPI maintenance therapy among 173 adult patients with relapsed/refractory NHL treated with a CD19/CD22 directed CAR-T product with or without autologous stem cell transplantation (ASCT) (139). In this study, CPI maintenance led to superior objective response rate (82.9% vs 60%; P = 0.04) and 2-year progression-free survival (59.8% vs 21.3%; P = 0.001), as well as longer CAR-T cell persistence, when compared to patients treated without CPI maintenance, suggesting that it a promising adjunctive strategy for future CAR-T trials.

### Bruton’s tyrosine kinase inhibition

Bruton’s tyrosine kinase (BTK) inhibitors target the B-cell receptor (BCR) signaling pathway, which normally plays a key role in B-cell activation and function (140). In cases of dysregulation, BTK can drive the proliferation of malignant B cells. For this reason, BTK inhibitors have been the mainstay of therapy for chronic lymphocytic leukemia and mantle cell lymphoma due to their B-cell depleting properties (141).

Interestingly, BTK inhibitors may amplify the efficacy of CAR-T cell therapy independently of their effects on B cells. The mechanism of this phenomenon is likely mediated via the inhibition of interleukin 2 (IL-2)-inducible T-cell kinase (ITK), which is a key downstream driver of Th2 immune signaling (142). In this way, blockade of ITK subverts Th2 immunity, thereby shifting to a Th1-based immune response, which is key for anti-tumor effect.

In a preclinical study utilizing tumor cultures and a xenograft lymphoma mouse model, co-administration of BTK inhibitors with CD19 CAR-T cells led to reduced T-cell exhaustion with tonic or recurrent antigen stimulation and improved *in vivo* persistence and anti-tumor efficacy (143). Mechanistically, BTK inhibitors suppressed CD3-ζ phosphorylation in both CARs and TCRs, leading to the downregulation of genes associated with T-cell activation signaling pathways, thus preventing detrimental hyperactivation and exhaustion.

Clinically, in a recent preliminary study of 37 patients receiving CD19 directed CAR-T therapy for relapsed/refractory B-cell lymphomas, 13 patients received co-administration of a BTK inhibitor in a non-randomized fashion. Although the results must be interpreted with caution, patients receiving BTK inhibitors had superior ORR (84.6% vs 66.7%), CR (61.5% vs 25.0%), and prolonged overall survival. Notably, CAR-T cells in the BTK inhibitor group demonstrated early differentiation and less exhaustion at three months (132). Similarly, a recent phase II trial of zanubrutinib administered as lead-in and maintenance therapy around CAR-T cell infusion in relapsed/refractory aggressive non-Hodgkin lymphoma was associated with increased circulating CD8+ T-cells and decreased frequencies of CD4+ regulatory T-cells and other exhausted phenotypes, and prolonged duration of clinical CR, compared with historical benchmarks of CAR-T monotherapy (144).

### Immunomodulatory drugs

Immunomodulatory drugs (IMiDs), including lenalidomide and pomalidomide, are a class of molecules derived from thalidomide, which have pleotropic anti-neoplastic effects via TNF-alpha-inhibitory, T-cell stimulatory and antiangiogenic activities (145). These effects are, in part, related to their effect on the cerebron ubiquitin ligase complex, whereby IMiD binding leads to selective ubiquitination and degradation of the Ikaros family transcriptional repressors, thus leading to increased T-cell activation and increased IL-2 production (146, 147). Furthermore, these agents directly counteract T-cell exhaustion by reducing suppressive Treg and PD-1^+^ T-cells and by inducing phosphorylation of the CD28 co-stimulatory domain and subsequently driving expression of NF-κB in T-cells, promoting survival (148–150). Given their T-cell stimulatory and anti-exhaustion effects, there has been strong interest in combining IMiDs with CAR-T cells to modulate clinical efficacy.

Preclinical studies have demonstrated improved CAR-T cell function when combined with lenalidomide. *In vitro* studies have demonstrated lenalidomide dependent enhancement of immunological synapse (IS) formation in CAR-T cells co-cultured with chronic lymphocytic leukemia cells (151).

Other studies have demonstrated that lenalidomide modulates CAR-T cell exhaustion via the upregulation of the pro-inflammatory cytokine IL-21. IL-21, which is negatively regulated by Ikaros, appears to have broad pro-activation and anti-exhaustion effects on native T-cells including alterations in cellular metabolism, decrease in checkpoint molecular expression and expansion of memory-like T-cells (152). Indeed CAR-T cells exposed to lenalidomide have demonstrated upregulation of IL-21, supporting the role of lenalidomide as a CAR-T modulating agent (153). Furthermore, lenalidomide dependent reversible *on and off switch* CAR’s have been developed, which may allow for reduced CAR-T cell exhaustion and improved safety via transient rest from CAR signaling while simultaneously harnessing the immunomodulatory effects of lenalidomide on CAR effector function (154).

Clinical data supporting the role of lenalidomide enhanced CAR-T cell therapy are limited. A pilot study of seven patients with relapsed/refractory DLBCL who received lenalidomide maintenance after CAR-T therapy demonstrated possible improvements in efficacy, with one patient demonstrating significant CAR-T re-expansion upon lenalidomide exposure. Further studies investigating the role of IMiDs in combination with CAR-T therapy are ongoing (NCT06762431, NCT04002401, NCT03070327).

Immunomodulatory agents (IMiDs) and their next-generation derivatives, cereblon E3 ligase modulators (CELMoDs), such as iberdomide and mezigdomide, have demonstrated the ability to reinvigorate T cell function by reversing the features of exhaustion (155, 156) Unlike conventional IMiDs, CELMoDs achieve deeper and faster degradation of transcription factors Aiolos and Ikaros (155). Clinical and preclinical studies have shown that iberdomide enhances T and NK cell proliferation, promotes effector memory phenotypes, and reduces inhibitory receptor expression without driving irreversible exhaustion (156). Similarly, mezigdomide has been shown to restore cytokine production pathways, reinvigorate exhausted T cells, and enhance anti-tumor activity in the context of bispecific antibody therapy (157). Mechanistically, these effects are attributed to epigenetic reprogramming and modulation of the immune microenvironment, making CELMoDs promising agents for overcoming immune dysfunction in relapsed or refractory multiple myeloma. Given these mechanisms, the potential combination of CELMoDs with CAR-T-cell therapy could also be explored in lymphoma, where CAR-T cell exhaustion similarly limits therapeutic efficacy.

### Epigenetic modification

The importance of epigenetic modifications, including DNA methylation, histone modifications, non-coding RNAs, and higher-order chromatin remodeling in T-cell exhaustion has been well demonstrated; therefore, mechanisms to manipulate CAR-T cell epigenetics have become an attractive mechanism to augment function (158).

Previous work has demonstrated that *de novo* DNA methylation promotes T-cell exhaustion and that inhibition of DNA methylation may enhance the effect of PDL-1 blockade on native T-cells (159). Furthermore, preclinical data have demonstrated that inhibition of DNMT3a, an enzyme which adds methyl groups to DNA, in T-cells can reduce exhaustion and enhance differentiation into a memory phenotype (160). Decitabine, an inhibitor of DNA methyltransferase, has been suggested as a possible means of enhancing CAR-T therapy by allowing for re-expression of naïve T cell associated genes that promote a memory cell phenotype, reducing expression of exhaustion associated genes, and enhancing cell proliferation (161). Indeed, CAR-T cells treated with decitabine demonstrated superior anti-tumor activity, and lymphoma cells pre-treated with decitabine were rendered more sensitive to CAR-T cell killing, in part by the enhancement of CD19 expression (162, 163).

Another approach involves targeting the histone methyltransferase EZH2, which mediates gene silencing via trimethylation of histone H3 lysine 27 (H3K27me3). In preclinical models, the use of tazemetostat, a selective EZH2 inhibitor, in combination with CAR-T cells, led to increased tumor immunogenicity in multiple cancer scenarios via enhancement of CAR-T cell activation, expansion, tumor infiltration and exhaustion reversal via mechanisms involving both CAR-T cells and target cancer cells (164). Clinical trials involving EZH2 inhibition to augment CAR-T cell therapy are currently ongoing.

Other early preclinical studies have demonstrated that histone acetyltransferase HDAC inhibitors and bromodomain and extra-terminal (BET) inhibitors may additionally be effective and counteract CAR-T cellular exhaustion and improve anti-tumor efficacy (165, 166).

### IL-4 inhibition

Recently, a comprehensive preclinical study utilized a genome-wide CRISPR knock-out screen to identify upstream regulators of CAR-T cell exhaustion, revealing interleukin-4 (IL-4) as a driver of CAR-T cell exhaustion (26). IL-4 exposure led to the rapid acquisition of exhaustion features and functional attenuation in CD8^+^ CAR-T, even in the absence of CD4^+^ T-cells, tumor cells or the tumor microenvironment. Finally, investigators demonstrated that therapeutic IL-4 blockade with a monoclonal antibody effectively improved T-cell persistence, anti-tumor effector functions and survival in a mantle cell lymphoma xenograft model. Collectively, this study highlights the IL-4/IL-4R pathway as a potent intrinsic driver of CAR-T exhaustion and the power of CRISPR-based platforms to uncover targetable drivers of CAR-T exhaustion.

### Modulation of TCF1

Other strategies for improving CAR-T efficacy have focused on modulating transcription factor expression and targeting inhibitory receptors. Enhancing the expression of transcription factors such as TCF1, which is involved in early T-cell development, can further help maintain a progenitor-like phenotype in T cells undergoing exhaustion (167, 168). Phosphoinositide 3-kinase (PI3K), like BTK, acts through the BCR signaling pathway to mediate normal cellular functioning, and dysregulation has been implicated in oncogenesis (169). PI3K inhibitor therapy has already demonstrated clinical efficacy in CLL and follicular lymphoma, though safety and toxicity profiles have limited their broader applicability (170). Preclinical research has demonstrated that ex vivo addition of the dual-PI3Kδ/γ inhibitor duvelisib during CAR-T cell manufacturing enhances *in vivo* expansion and anti-tumor activity against CLL. The underlying mechanism is thought to be increased TCF1 expression driving stem-like behavior and enhances persistence and survival (171). Similarly, addition of the tyrosine kinase inhibitor dasatinib during ex vivo expansion led to enhanced therapeutic efficacy and *in vivo* persistence, in part due to the upregulation of TCF1 (172). These findings establish TCF1 as a key regulator of CAR-T cell performance, with its modulation offering a promising avenue to overcome exhaustion and improve therapeutic outcomes.

### CMV-specific CD19 CAR-T cells and CMV vaccines

Another innovative strategy under development is the generation of CMV-specific CD19 directed CAR T-cells. These cells are developed through CMV-seropositive donor peripheral blood mononuclear cells that were stimulated with the CMVpp65 protein to enrich for CMV-reactive T-cells, followed by expansion and transduction with the CD19 CAR vector. To further enhance response and promote proliferation, recipients of these cells subsequently receive CMV-based vaccinations to promote antigen-driven restimulation through the native TCR, thereby promoting an *in vivo* proliferative response. In preclinical studies, these cells exhibited strong anti-tumor activity and persistence. The manufactured products demonstrated a favorable phenotype with enrichment of memory-associated markers and lower baseline expression of exhaustion markers (173). Currently, early clinical trials are underway to investigate this platform for intermediate/high-grade relapsed/refractory B cell lymphoma and following stem cell transplant to prevent disease recurrence (174–176). Beyond its beneficial effects on persistence, these cells offer unique potential for post-infusion boosting via CMV vaccination, which may have applications in the setting of relapsed or persistent disease.

### Manufacturing strategies

Additional strategies for improving CAR-T cell fitness have focused on optimizing CAR-T cell manufacturing. Mounting evidence suggests that multipotent T memory stem cells (T_SCM_) in the CAR-T product are responsible for superior CAR-T cell activity, due to their greater capacity for expansion, resistance to exhaustion and long-term persistence (177, 178). Moreover, improved *in vivo* expansion and anti-tumor responses have been observed with CAR-T cell products expressing genes associated with early memory states of differentiation, such as *TCF7* and *LEF1* (179, 180). In a study of 54 patients undergoing CD22-directed CAR-T cell therapy, apheresis and infusion products were compared between those who achieved complete remission and those who did not. Non-responders were more likely to have been given CAR-T cells that were terminally differentiated and express CD62L, a biomarker associated with endothelial cell adhesion and entry into secondary lymphoid structures, while responders were more likely to express the IL-7 receptor α (CD127) on CD4^+^ T cells in the starting apheresis cells, which is associated with an early memory phenotype (181).

In accordance with these findings, another study developed a protocol to manufacture a more progenitor/stem-like CAR-T cell product by culturing naïve-enriched T cells in the presence of IL-15 and IL-7. This stem-like CAR-T product exhibited a higher CD4:CD8 ratio, which led to increased proliferative capacity, cytotoxicity, cytokine production, survival, and tumor control in mice, especially in combination with a PD-1-blocking antibody (182). Another strategy involving activation of naïve T-cells in the presence of IL-7, IL-21, and a glycogen synthase-3β inhibitor allowed for the generation of significant numbers of CD8^+^ T_SCM_ exhibiting improved metabolic fitness and robust *in vivo* activity in acute lymphoblastic leukemia xenografts (183). Clinical studies utilizing T_SCM_ selected CAR-T products are ongoing.

## Conclusion

**Figure 4:**
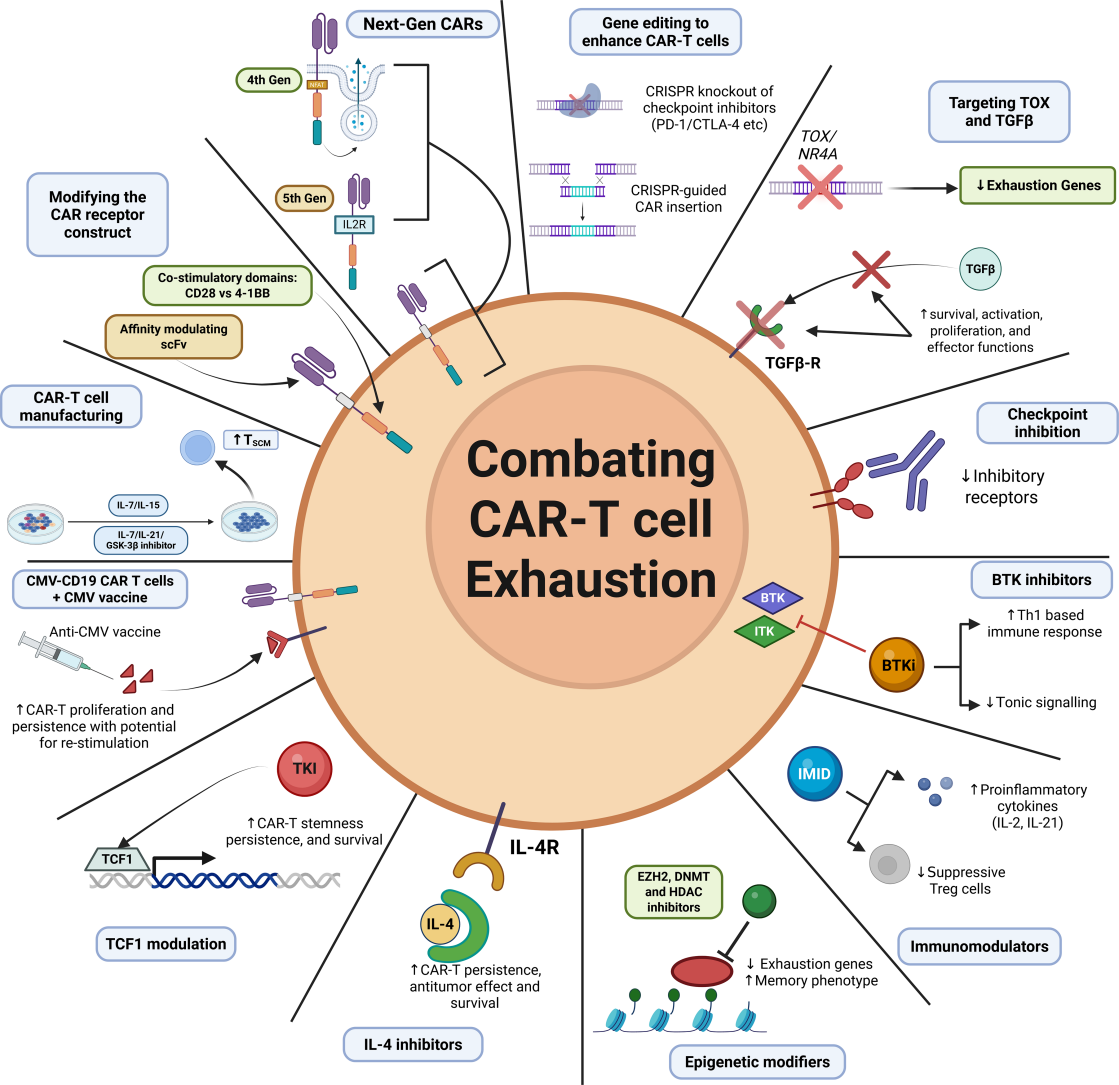
Strategies to combat CAR-T cell exhaustion CAR-T cell exhaustion is a major contributor to therapeutic failure. Numerous strategies are under development to combat exhaustion, including modification of the CAR construct, adjunctive therapies and manufacturing strategies. Figure created in https://BioRender.com.

CAR-T cell therapy has transformed the treatment landscape for lymphoid malignancies in the last decade, offering opportunities for cure in otherwise treatment refractory diseases; however, despite its success, most patients treated with CAR-T therapy will ultimately relapse. CAR-T cell exhaustion has been identified as a major contributing factor to treatment failure. Analogous to native T-cells, CAR-T cell exhaustion arises in the setting of persistent CAR stimulation, as well as intrinsic signaling within CAR constructs, whereby molecular and epigenetic pathways are activated, resulting in impaired anti-cancer efficacy. Advances in our understanding of CAR-T cell exhaustion have allowed for rational modifications in CAR design, such as affinity modulation of the scFv antigen-binding domain, alterations of co-stimulatory domains, and CRISPR-mediated editing of exhaustion-related molecular pathways, which have shown promise in reducing exhaustions while maintaining anti-cancer efficacy. Furthermore, several adjunctive agents, such as checkpoint inhibitors, BTK inhibitors, IMiDs, and epigenetic modifiers, have demonstrated promise in modulating CAR-T cell function through reduced exhaustion. Improving the manufacturing strategies can also augment the quality of CAR-T cell therapies (**Figure 4**). Although further research is needed to fully elucidate the mechanisms underlying CAR-T exhaustion in the context of hematologic malignancies, the strategies discussed above hold promise to broaden the clinical impact and durability of CAR-T cell therapy.

## References

[1] Bhaskar ST Dholaria B Savani BN Sengsayadeth S Oluwole O . Overview of approved CAR-T products and utility in clinical practice. Clin Hematol Int. (2024) 6:93–9. doi: 10.46989/001c.124277. PMID: 39469119 PMC11514108

[2] Sterner RC Sterner RM . CAR-T cell therapy: current limitations and potential strategies. Blood Cancer J. (2021) 11:69. doi: 10.1038/s41408-021-00459-7. PMID: 33824268 PMC8024391

[3] Kuwana Y Asakura Y Utsunomiya N Nakanishi M Arata Y Itoh S . Expression of chimeric receptor composed of immunoglobulin-derived V regions and T-cell receptor-derived C regions. Biochem Biophys Res Commun. (1987) 149:960–8. doi: 10.1016/0006-291x(87)90502-x. PMID: 3122749

[4] Kalos M Levine BL Porter DL Katz S Grupp SA Bagg A . T cells with chimeric antigen receptors have potent antitumor effects and can establish memory in patients with advanced leukemia. Sci Transl Med. (2011) 3:95ra73. doi: 10.1126/scitranslmed.3002842. PMID: 21832238 PMC3393096

[5] Honikel MM Olejniczak SH . Co-stimulatory receptor signaling in CAR-T cells. Biomolecules. (2022) 12. doi: 10.3390/biom12091303. PMID: 36139142 PMC9496564

[6] Imai C Mihara K Andreansky M Nicholson IC Pui C-H Geiger TL . Chimeric receptors with 4-1BB signaling capacity provoke potent cytotoxicity against acute lymphoblastic leukemia. Leukemia. (2004) 18:676–84. doi: 10.1038/sj.leu.2403302. PMID: 14961035

[7] Savoldo B Ramos CA Liu E Mims MP Keating MJ Carrum G . CD28 costimulation improves expansion and persistence of chimeric antigen receptor-modified T cells in lymphoma patients. J Clin Invest. (2011) 121:1822–6. doi: 10.1172/JCI46110. PMID: 21540550 PMC3083795

[8] Schuster SJ Tam CS Borchmann P Worel N McGuirk JP Holte H . Long-term clinical outcomes of tisagenlecleucel in patients with relapsed or refractory aggressive B-cell lymphomas (JULIET): a multicentre, open-label, single-arm, phase 2 study. Lancet Oncol. (2021) 22:1403–15. doi: 10.1016/S1470-2045(21)00375-2. PMID: 34516954

[9] Fowler NH Dickinson M Dreyling M Martinez-Lopez J Kolstad A Butler J . Tisagenlecleucel in adult relapsed or refractory follicular lymphoma: the phase 2 ELARA trial. Nat Med. (2022) 28:325–32. doi: 10.1038/s41591-021-01622-0. PMID: 34921238

[10] Dreyling M Fowler NH Dickinson M Martinez-Lopez J Kolstad A Butler J . Durable response after tisagenlecleucel in adults with relapsed/refractory follicular lymphoma: ELARA trial update. Blood. (2024) 143:1713–25. doi: 10.1182/blood.2023021567. PMID: 38194692 PMC11103095

[11] Neelapu SS Locke FL Bartlett NL Lekakis LJ Miklos DB Jacobson CA . Axicabtagene ciloleucel CAR T-cell therapy in refractory large B-cell lymphoma. N Engl J Med. (2017) 377:2531–44. doi: 10.1056/NEJMoa1707447. PMID: 29226797 PMC5882485

[12] Neelapu SS Jacobson CA Ghobadi A Miklos DB Lekakis LJ Oluwole OO . 5-year follow-up supports curative potential of axicabtagene ciloleucel in refractory large B-cell lymphoma (ZUMA-1). Blood. (2023) 141(19):2307–2315. doi: 10.1182/blood.2022018893. PMID: 36821768 PMC10646788

[13] Locke FL Miklos DB Jacobson CA Perales M-A Kersten M-J Oluwole OO . Axicabtagene ciloleucel as second-line therapy for large B-cell lymphoma. N Engl J Med. (2022) 386:640–54. doi: 10.1056/NEJMoa2116133. PMID: 34891224

[14] Jacobson CA Chavez JC Sehgal AR William BM Munoz J Salles G . Axicabtagene ciloleucel in relapsed or refractory indolent non-Hodgkin lymphoma (ZUMA-5): a single-arm, multicentre, phase 2 trial. Lancet Oncol. (2022) 23:91–103. doi: 10.1016/S1470-2045(21)00591-X. PMID: 34895487

[15] Abramson JS Palomba ML Gordon LI Lunning M Wang M Arnason J . Two-year follow-up of lisocabtagene maraleucel in relapsed or refractory large B-cell lymphoma in TRANSCEND NHL 001. Blood. (2024) 143:404–16. doi: 10.1182/blood.2023020854. PMID: 37890149

[16] Abramson JS Solomon SR Arnason J Johnston PB Glass B Bachanova V . Lisocabtagene maraleucel as second-line therapy for large B-cell lymphoma: primary analysis of the phase 3 TRANSFORM study. Blood. (2023) 141:1675–84. doi: 10.1182/blood.2022018730. PMID: 36542826 PMC10646768

[17] Kamdar MK Solomon SR Arnason J Johnston PB Glaß B Bachanova V . Lisocabtagene maraleucel (liso-cel) vs standard of care (SOC) with salvage chemotherapy (CT) followed by autologous stem cell transplantation (ASCT) as second-line (2L) treatment in patients (pt) with R/R large B-cell lymphoma (LBCL): 3-year follow-up (FU. J Clin Oncol. (2024) 42:7013. doi: 10.1200/JCO.2024.42.16_suppl.7013. PMID: 41735675

[18] Morschhauser F Dahiya S Palomba ML Martin Garcia-Sancho A Reguera Ortega JL Kuruvilla J . Lisocabtagene maraleucel in follicular lymphoma: the phase 2 TRANSCEND FL study. Nat Med. (2024) 30:2199–207. doi: 10.1038/s41591-024-02986-9. PMID: 38830991 PMC11333271

[19] Wang M Siddiqi T Gordon LI Kamdar M Lunning M Hirayama AV . Lisocabtagene maraleucel in relapsed/refractory mantle cell lymphoma: primary analysis of the mantle cell lymphoma cohort from TRANSCEND NHL 001, a phase I multicenter seamless design study. J Clin Oncol. (2024) 42:1146–57. doi: 10.1200/JCO.23.02214. PMID: 38072625 PMC11741176

[20] Siddiqi T Maloney DG Kenderian SS Brander DM Dorritie K Soumerai J . Lisocabtagene maraleucel in chronic lymphocytic leukaemia and small lymphocytic lymphoma (TRANSCEND CLL 004): a multicentre, open-label, single-arm, phase 1–2 study. Lancet. (2023) 402:641–54. doi: 10.1016/S0140-6736(23)01052-8. PMID: 37295445 PMC11753452

[21] Palomba ML Schuster SJ Karmali R Skarbnik AP Abramson JS Ardeshna K . Lisocabtagene maraleucel in patients with relapsed or refractory marginal zone lymphoma (TRANSCEND FL): primary analysis results from the global, multicohort, single-arm, phase 2 study. Lancet. (2026) 407(10532):963–75. doi: 10.1016/S0140-6736(25)02435-3, PMID: 41692020

[22] Wang M Munoz J Goy A Locke FL Jacobson CA Hill BT . KTE-X19 CAR T-cell therapy in relapsed or refractory mantle-cell lymphoma. N Engl J Med. (2020) 382:1331–42. doi: 10.1056/NEJMoa1914347. PMID: 32242358 PMC7731441

[23] Wang M Munoz J Goy A Locke FL Jacobson CA Hill BT . Three-year follow-up of KTE-X19 in patients with relapsed/refractory mantle cell lymphoma, including high-risk subgroups, in the ZUMA-2 study. J Clin Oncol. (2023) 41:555–67. doi: 10.1200/JCO.21.02370. PMID: 35658525 PMC9870225

[24] Di Blasi R Le Gouill S Bachy E Cartron G Beauvais D Le Bras F . Outcomes of patients with aggressive B-cell lymphoma after failure of anti-CD19 CAR T-cell therapy: a DESCAR-T analysis. Blood. (2022) 140:2584–93. doi: 10.1182/blood.2022016945. PMID: 36122385

[25] Alarcon Tomas A Fein JA Fried S Flynn JR Devlin SM Fingrut WB . Outcomes of first therapy after CD19-CAR-T treatment failure in large B-cell lymphoma. Leukemia. (2023) 37:154–63. doi: 10.1038/s41375-022-01739-2. PMID: 36335261 PMC9892211

[26] Stewart CM Siegler EL Sakemura RL Cox MJ Huynh T Kimball B . IL-4 drives exhaustion of CD8+ CART cells. Nat Commun. (2024) 15:7921. doi: 10.1038/s41467-024-51978-3. PMID: 39266501 PMC11393358

[27] Chow VA Gopal AK Maloney DG Turtle CJ Smith SD Ujjani CS . Outcomes of patients with large B‐cell lymphomas and progressive disease following CD19‐specific CAR T‐cell therapy. Am J Hematol. (2019) 94(8):E209–E213. doi: 10.1002/ajh.25505. PMID: 31056762 PMC6776079

[28] Sworder BJ Kurtz DM Alig SK Frank MJ Shukla N Garofalo A . Determinants of resistance to engineered T cell therapies targeting CD19 in large B cell lymphomas. Cancer Cell. (2023) 41:210–225.e5. doi: 10.1016/j.ccell.2022.12.005. PMID: 36584673 PMC10010070

[29] Shah NN Fry TJ . Mechanisms of resistance to CAR T cell therapy. Nat Rev Clin Oncol. (2019) 16(6):372–385. doi: 10.1038/s41571-019-0184-6. PMID: 30837712 PMC8214555

[30] Xu X Sun Q Liang X Chen Z Zhang X Zhou X . Mechanisms of relapse after CD19 CAR T-cell therapy for acute lymphoblastic leukemia and its prevention and treatment strategies. Front Immunol. (2019) 10:2664. doi: 10.3389/fimmu.2019.02664. PMID: 31798590 PMC6863137

[31] Long AH Haso WM Shern JF Wanhainen KM Murgai M Ingaramo M . 4-1BB costimulation ameliorates T cell exhaustion induced by tonic signaling of chimeric antigen receptors. Nat Med. (2015) 21:581–90. doi: 10.1038/nm.3838. PMID: 25939063 PMC4458184

[32] Baessler A Vignali DAA . T cell exhaustion. Annu Rev Immunol. (2024) 42:179–206. doi: 10.1146/annurev-immunol-090222-110914. PMID: 38166256

[33] Cui W Kaech SM . Generation of effector CD8+ T cells and their conversion to memory T cells. Immunol Rev. (2010) 236:151–66. doi: 10.1111/j.1600-065X.2010.00926.x. PMID: 20636815 PMC4380273

[34] Moskophidis D Lechner F Pircher H Zinkernagel RM . Virus persistence in acutely infected immunocompetent mice by exhaustion of antiviral cytotoxic effector T cells. Nature. (1993) 362:758–61. doi: 10.1038/362758a0. PMID: 8469287

[35] Zajac AJ Blattman JN Murali-Krishna K Sourdive DJ Suresh M Altman JD . Viral immune evasion due to persistence of activated T cells without effector function. J Exp Med. (1998) 188:2205–13. doi: 10.1084/jem.188.12.2205. PMID: 9858507 PMC2212420

[36] Ahn T Bae E-A Seo H . Decoding and overcoming T cell exhaustion: Epigenetic and transcriptional dynamics in CAR-T cells against solid tumors. Mol Ther. (2024) 32:1617–27. doi: 10.1016/j.ymthe.2024.04.004. PMID: 38582965 PMC11184340

[37] Yi JS Cox MA Zajac AJ . T-cell exhaustion: characteristics, causes and conversion. Immunology. (2010) 129:474–81. doi: 10.1111/j.1365-2567.2010.03255.x. PMID: 20201977 PMC2842494

[38] Scharping NE Rivadeneira DB Menk AV Vignali PDA Ford BR Rittenhouse NL . Mitochondrial stress induced by continuous stimulation under hypoxia rapidly drives T cell exhaustion. Nat Immunol. (2021) 22:205–15. doi: 10.1038/s41590-020-00834-9. PMID: 33398183 PMC7971090

[39] Zhang J Lyu T Cao Y Feng H . Role of TCF‐1 in differentiation, exhaustion, and memory of CD8 + T cells: a review. FASEB J. (2021) 35. doi: 10.1096/fj.202002566R. PMID: 33913198

[40] Jiang Y Li Y Zhu B . T-cell exhaustion in the tumor microenvironment. Cell Death Dis. (2015) 6:e1792. doi: 10.1038/cddis.2015.162. PMID: 26086965 PMC4669840

[41] Kallingal A Olszewski M Maciejewska N Brankiewicz W Baginski M . Cancer immune escape: the role of antigen presentation machinery. J Cancer Res Clin Oncol. (2023) 149:8131–41. doi: 10.1007/s00432-023-04737-8. PMID: 37031434 PMC10374767

[42] Yi M Li T Niu M Zhang H Wu Y Wu K . Targeting cytokine and chemokine signaling pathways for cancer therapy. Signal Transduct Target Ther. (2024) 9:176. doi: 10.1038/s41392-024-01868-3. PMID: 39034318 PMC11275440

[43] Cavazzoni A Digiacomo G . Role of cytokines and other soluble factors in tumor development: rationale for new therapeutic strategies. Cells. (2023) 12:2532. doi: 10.3390/cells12212532. PMID: 37947610 PMC10647643

[44] Mirlekar B . Tumor promoting roles of IL-10, TGF-β, IL-4, and IL-35: its implications in cancer immunotherapy. SAGE Open Med. (2022) 10. doi: 10.1177/20503121211069012. PMID: 35096390 PMC8793114

[45] Heidari-Foroozan M Rezalotfi A Rezaei N . The molecular landscape of T cell exhaustion in the tumor microenvironment and reinvigoration strategies. Int Rev Immunol. (2024) 43:419–40. doi: 10.1080/08830185.2024.2401352. PMID: 39257319

[46] Nixon BG Kuo F Ji L Liu M Capistrano K Do M . Tumor-associated macrophages expressing the transcription factor IRF8 promote T cell exhaustion in cancer. Immunity. (2022) 55:2044–2058.e5. doi: 10.1016/j.immuni.2022.10.002. PMID: 36288724 PMC9649891

[47] Kersten K Hu KH Combes AJ Samad B Harwin T Ray A . Spatiotemporal co-dependency between macrophages and exhausted CD8+ T cells in cancer. Cancer Cell. (2022) 40:624–638.e9. doi: 10.1016/j.ccell.2022.05.004. PMID: 35623342 PMC9197962

[48] Liu Y-N Yang J-F Huang D-J Ni H-H Zhang C-X Zhang L . Hypoxia induces mitochondrial defect that promotes T cell exhaustion in tumor microenvironment through MYC-regulated pathways. Front Immunol. (2020) 11:1906. doi: 10.3389/fimmu.2020.01906. PMID: 32973789 PMC7472844

[49] Jayaraman J Mellody MP Hou AJ Desai RP Fung AW Pham AHT . CAR-T design: elements and their synergistic function. EBioMedicine. (2020) 58:102931. doi: 10.1016/j.ebiom.2020.102931. PMID: 32739874 PMC7393540

[50] Salter AI Rajan A Kennedy JJ Ivey RG Shelby SA Leung I . Comparative analysis of TCR and CAR signaling informs CAR designs with superior antigen sensitivity and *in vivo* function. Sci Signal. (2021) 14. doi: 10.1126/scisignal.abe2606. PMID: 34429382 PMC8613804

[51] Jiang P Zhang Z Hu Y Liang Z Han Y Li X . Single-cell ATAC-seq maps the comprehensive and dynamic chromatin accessibility landscape of CAR-T cell dysfunction. Leukemia. (2022) 36:2656–68. doi: 10.1038/s41375-022-01676-0. PMID: 35962059

[52] Bartleson JM Viehmann Milam AA Donermeyer DL Horvath S Xia Y Egawa T . Strength of tonic T cell receptor signaling instructs T follicular helper cell-fate decisions. Nat Immunol. (2020) 21:1384–96. doi: 10.1038/s41590-020-0781-7. PMID: 32989327 PMC7578106

[53] Chen J Qiu S Li W Wang K Zhang Y Yang H . Tuning charge density of chimeric antigen receptor optimizes tonic signaling and CAR-T cell fitness. Cell Res. (2023) 33:341–54. doi: 10.1038/s41422-023-00789-0. PMID: 36882513 PMC10156745

[54] Landoni E Fucá G Wang J Chirasani VR Yao Z Dukhovlinova E . Modifications to the framework regions eliminate chimeric antigen receptor tonic signaling. Cancer Immunol Res. (2021) 9:441–53. doi: 10.1158/2326-6066.CIR-20-0451. PMID: 33547226 PMC8137530

[55] Beider K Itzhaki O Schachter J Grushchenko-Polaq AH Voevoda-Dimenshtein V Rosenberg E . Molecular and functional signatures associated with CAR T cell exhaustion and impaired clinical response in patients with B cell Malignancies. Cells. (2022) 11. doi: 10.3390/cells11071140. PMID: 35406703 PMC8997745

[56] Chow A Perica K Klebanoff CA Wolchok JD . Clinical implications of T cell exhaustion for cancer immunotherapy. Nat Rev Clin Oncol. (2022) 19:775–90. doi: 10.1038/s41571-022-00689-z. PMID: 36216928 PMC10984554

[57] Cappell KM Kochenderfer JN . A comparison of chimeric antigen receptors containing CD28 versus 4-1BB costimulatory domains. Nat Rev Clin Oncol. (2021) 18:715–27. doi: 10.1038/s41571-021-00530-z. PMID: 34230645

[58] Acharya S Basar R Daher M Rafei H Li P Uprety N . CD28 costimulation augments CAR signaling in NK cells via the LCK/CD3ζ/ZAP70 signaling axis. Cancer Discov. (2024) 14:1879–900. doi: 10.1158/2159-8290.CD-24-0096. PMID: 38900051 PMC11452288

[59] Frigault MJ Lee J Basil MC Carpenito C Motohashi S Scholler J . Identification of chimeric antigen receptors that mediate constitutive or inducible proliferation of T cells. Cancer Immunol Res. (2015) 3:356–67. doi: 10.1158/2326-6066.CIR-14-0186. PMID: 25600436 PMC4390458

[60] Sun C Shou P Du H Hirabayashi K Chen Y Herring LE . THEMIS-SHP1 recruitment by 4-1BB tunes LCK-mediated priming of chimeric antigen receptor-redirected T cells. Cancer Cell. (2020) 37:216–225.e6. doi: 10.1016/j.ccell.2019.12.014. PMID: 32004441 PMC7397569

[61] Philipson BI O’Connor RS May MJ June CH Albelda SM Milone MC . 4-1BB costimulation promotes CAR T cell survival through noncanonical NF-κB signaling. Sci Signal. (2020) 13. doi: 10.1126/scisignal.aay8248. PMID: 32234960 PMC7883633

[62] Wu L Chen J Cai R Wang X Liu Y Zheng Q . Difference in efficacy and safety of anti-CD19 chimeric antigen receptor T-cell therapy containing 4-1BB and CD28 co-stimulatory domains for B-cell acute lymphoblastic leukemia. Cancers (Basel). (2023) 15. doi: 10.3390/cancers15102767. PMID: 37345104 PMC10216493

[63] Maloney DG Kuruvilla J Liu FF Kostic A Kim Y Bonner A . Matching-adjusted indirect treatment comparison of liso-cel versus axi-cel in relapsed or refractory large B cell lymphoma. J Hematol Oncol. (2021) 14:140. doi: 10.1186/s13045-021-01144-9. PMID: 34493319 PMC8425084

[64] Oluwole OO Chen JM Chan K Patel AR Jansen JP Keeping S . Matching-adjusted indirect comparison of axi-cel and liso-cel in relapsed or refractory large B-cell lymphoma. Leuk Lymphoma. (2022) 63:3052–62. doi: 10.1080/10428194.2022.2113526. PMID: 36048026

[65] Abramson JS Kamdar M Liu FF Crotta A Previtali A Klijn SL . Matching-adjusted indirect comparison (MAIC) of lisocabtagene maraleucel (liso-cel) versus axicabtagene ciloleucel (axi-cel) for second-line (2L) treatment of patients (pts) with refractory/early relapsed (R/R) large B-cell lymphoma (LBCL): Update with 34. Blood. (2024) 144:3130. doi: 10.1182/blood-2024-200937. PMID: 41761659

[66] Boardman AP Reguera JL Wang P Ellis J Bar M Kumar J . Matching-adjusted indirect comparison (MAIC) of lisocabtagene maraleucel (liso-cel) versus axicabtagene ciloleucel (axi-cel) and tisagenlecleucel (tisa-cel) for treatment of third-line or later (3L+) R/R follicular lymphoma (FL): Update with 24 months of. J Clin Oncol. (2025) 43. doi: 10.1200/JCO.2025.43.16_suppl.e19049. PMID: 40980778

[67] Meng J Wu X Sun Z Xun R Liu M Hu R . Efficacy and safety of CAR-T cell products axicabtagene ciloleucel, tisagenlecleucel, and lisocabtagene maraleucel for the treatment of hematologic Malignancies: A systematic review and meta-analysis. Front Oncol. (2021) 11:698607. doi: 10.3389/fonc.2021.698607. PMID: 34381720 PMC8350577

[68] Tun AM Patel R St-Pierre F Ouchveridze E Niu A Obasi J . Chimeric antigen receptor T-cell therapy in elderly patients with relapsed or refractory large B-cell lymphoma: A multicenter study. Blood. (2023) 142:311. doi: 10.1182/blood-2023-179509. PMID: 41761659

[69] Portuguese AJ Huang JJ Jeon Y Taheri M Albittar A Liang EC . Real-world comparison of lisocabtagene maraleucel and axicabtagene ciloleucel in large B-cell lymphoma: An inverse probability of treatment weighting analysis with 3-year follow up. Haematologica. (2025) 9(3):455–462. doi: 10.3324/haematol.2024.287010. PMID: 40079097 PMC12399936

[70] Looka A Qualls DA Matthews D Redd RA Sakellis C Duffy C . A real-world comparison of commercial-use axicabtagene ciloleucel and lisocabtagene maraleucel in large B-cell lymphoma. Blood Adv. (2025) 9:455–62. doi: 10.1182/bloodadvances.2024012992. PMID: 39546746 PMC11808612

[71] Yu S Abdulhaleem M Safi SUD . Real World Comparison of Axicabtagene Ciloleucel and Lisocabtagene Maraleucel in Relapsed or Refractory Diffuse Large B-Cell Lymphoma. Blood (2024) 144(Supplement 1):4493. doi: 10.1182/blood-2024-194887

[72] Ali A Ahmed N Kim S Bye M Mirza S Sieg AG . Real world comparison of efficacy and safety of fludarabine-versus bendamustine-based lymphodepleting chemotherapy for cd19 chimeric antigen receptor (car) t-cell therapy in relapse/refractory (r/r) large b-cell lymphoma (LBCL). Blood. (2024) 144(Supplement 1):71. doi: 10.1182/blood-2024-200269

[73] Seo H Chen J González-Avalos E Samaniego-Castruita D Das A Wang YH . TOX and TOX2 transcription factors cooperate with NR4A transcription factors to impose CD8+ T cell exhaustion. Proc Natl Acad Sci USA. (2019) 116:12410–5. doi: 10.1073/pnas.1905675116. PMID: 31152140 PMC6589758

[74] Zhang X Zhang C Qiao M Cheng C Tang N Lu S . Depletion of BATF in CAR-T cells enhances antitumor activity by inducing resistance against exhaustion and formation of central memory cells. Cancer Cell. (2022) 40:1407–1422.e7. doi: 10.1016/j.ccell.2022.09.013. PMID: 36240777

[75] García-Calderón CB Sierro-Martínez B García-Guerrero E Sanoja-Flores L Muñoz-García R Ruiz-Maldonado V . Monitoring of kinetics and exhaustion markers of circulating CAR-T cells as early predictive factors in patients with B-cell Malignancies. Front Immunol. (2023) 14:1152498. doi: 10.3389/fimmu.2023.1152498. PMID: 37122702 PMC10140355

[76] Ventin M Cattaneo G Maggs L Arya S Wang X Ferrone CR . Implications of high tumor burden on chimeric antigen receptor T-cell immunotherapy: A review. JAMA Oncol. (2024) 10:115–21. doi: 10.1001/jamaoncol.2023.4504. PMID: 37943567

[77] Ponzo M Drufuca L Buracchi C Sindoni MM Nucera S Bugarin C . Acquisition of an immunosuppressive microenvironment after anti-CD19 CAR T-cell therapy is associated with T-cell dysfunction and resistance. J Immunother Cancer. (2025) 13. doi: 10.1136/jitc-2025-011768. PMID: 41135951 PMC12557794

[78] Scholler N Perbost R Locke FL Jain MD Turcan S Danan C . Tumor immune contexture is a determinant of anti-CD19 CAR T cell efficacy in large B cell lymphoma. Nat Med. (2022) 28:1872–82. doi: 10.1038/s41591-022-01916-x. PMID: 36038629 PMC9499856

[79] Chou J Plaks V Poddar S Wang Z Locke FL Neelapu SS . Favorable tumor immune microenvironment (TME) and robust chimeric antigen receptor (CAR) T-cell expansion may overcome tumor burden (TB) and promote durable efficacy with axicabtagene ciloleucel (axi-cel) in large B-cell lymphoma (LBCL). J Clin Oncol. (2021) 39:7536. doi: 10.1200/JCO.2021.39.15_suppl.7536. PMID: 41735675

[80] Li W Pan X Chen L Cui H Mo S Pan Y . Cell metabolism-based optimization strategy of CAR-T cell function in cancer therapy. Front Immunol. (2023) 14:1186383. doi: 10.3389/fimmu.2023.1186383. PMID: 37342333 PMC10278966

[81] Rivadeneira DB Thosar S Quann K Gunn WG Dean VG Xie B . Oxidative-stress-induced telomere instability drives T cell dysfunction in cancer. Immunity. (2025) 58:2524–2540.e5. doi: 10.1016/j.immuni.2025.08.008. PMID: 40930086 PMC13059126

[82] Peralta RM Xie B Lontos K Nieves-Rosado H Spahr K Joshi S . Dysfunction of exhausted T cells is enforced by MCT11-mediated lactate metabolism. Nat Immunol. (2024) 25:2297–307. doi: 10.1038/s41590-024-01999-3. PMID: 39516648 PMC11588660

[83] Yan Z-X Dong Y Qiao N Zhang Y-L Wu W Zhu Y . Cholesterol efflux from C1QB-expressing macrophages is associated with resistance to chimeric antigen receptor T cell therapy in primary refractory diffuse large B cell lymphoma. Nat Commun. (2024) 15:5183. doi: 10.1038/s41467-024-49495-4. PMID: 38890370 PMC11189439

[84] Jin J Lin L Meng J Jiang L Zhang M Fang Y . High-multiplex single-cell imaging analysis reveals tumor immune contexture associated with clinical outcomes after CAR T cell therapy. Mol Ther. (2024) 32:1252–65. doi: 10.1016/j.ymthe.2024.03.023. PMID: 38504519 PMC11081919

[85] Stahl D Gödel P Balke-Want H Gholamipoorfard R Segbers P Tetenborg L . CSF1R+ myeloid-monocytic cells drive CAR-T cell resistance in aggressive B cell lymphoma. Cancer Cell. (2025) 43:1476–1494.e10. doi: 10.1016/j.ccell.2025.05.013. PMID: 40513575

[86] Schmid DA Irving MB Posevitz V Hebeisen M Posevitz-Fejfar A Sarria J-C . Evidence for a TCR affinity threshold delimiting maximal CD8 T cell function. J Immunol. (2010) 184:4936–46. doi: 10.4049/jimmunol.1000173. PMID: 20351194

[87] Vander Mause ER Atanackovic D Lim CS Luetkens T . Roadmap to affinity-tuned antibodies for enhanced chimeric antigen receptor T cell function and selectivity. Trends Biotechnol. (2022) 40:875–90. doi: 10.1016/j.tibtech.2021.12.009. PMID: 35078657

[88] Ghorashian S Kramer AM Onuoha S Wright G Bartram J Richardson R . Enhanced CAR T cell expansion and prolonged persistence in pediatric patients with ALL treated with a low-affinity CD19 CAR. Nat Med. (2019) 25:1408–14. doi: 10.1038/s41591-019-0549-5. PMID: 31477906

[89] Roddie C Sandhu KS Tholouli E Logan AC Shaughnessy P Barba P . Obecabtagene autoleucel in adults with B-cell acute lymphoblastic leukemia. N Engl J Med. (2024) 391:2219–30. doi: 10.1056/NEJMoa2406526. PMID: 39602653 PMC12818175

[90] Esquinas E Moreno-Sanz A Sandá V Stodulski-Ciesla D Borregón J Peña-Blanque V . Preclinical development of three novel CARs targeting CD79b for the treatment of non-Hodgkin’s lymphoma and characterization of the loss of the target antigen. J Immunother Cancer. (2024) 12. doi: 10.1136/jitc-2024-009485. PMID: 39694704 PMC11667269

[91] Feucht J Sun J Eyquem J Ho Y-J Zhao Z Leibold J . Calibration of CAR activation potential directs alternative T cell fates and therapeutic potency. Nat Med. (2019) 25:82–8. doi: 10.1038/s41591-018-0290-5. PMID: 30559421 PMC6532069

[92] Boucher JC Li G Kotani H Cabral ML Morrissey D Lee SB . CD28 costimulatory domain-targeted mutations enhance chimeric antigen receptor T-cell function. Cancer Immunol Res. (2021) 9:62–74. doi: 10.1158/2326-6066.CIR-20-0253. PMID: 33188139 PMC7864379

[93] Park JH Palomba ML Perica K Devlin SM Shah G Dahi PB . Results from first-in-human phase I study of a novel CD19-1XX chimeric antigen receptor with calibrated signaling in large B-cell lymphoma. J Clin Oncol. (2025) 43:2418–28. doi: 10.1200/JCO-24-02424. PMID: 39883889 PMC12270773

[94] Drent E Poels R Ruiter R van de Donk NWCJ Zweegman S Yuan H . Combined CD28 and 4-1BB costimulation potentiates affinity-tuned chimeric antigen receptor–engineered T cells. Clin Cancer Res. (2019) 25:4014–25. doi: 10.1158/1078-0432.CCR-18-2559. PMID: 30979735 PMC7477921

[95] Roselli E Boucher JC Li G Kotani H Spitler K Reid K . 4-1BB and optimized CD28 co-stimulation enhances function of human mono-specific and bi-specific third-generation CAR T cells. J Immunother Cancer. (2021) 9. doi: 10.1136/jitc-2021-003354. PMID: 34706886 PMC8552146

[96] Derigs P Schubert M-L Dreger P Schmitt A Yousefian S Haas S . Third-generation anti-CD19 CAR T cells for relapsed/refractory chronic lymphocytic leukemia: A phase 1/2 study. Leukemia. (2024) 38:2419–28. doi: 10.1038/s41375-024-02392-7. PMID: 39192036 PMC11519001

[97] Ramos CA Rouce R Robertson CS Reyna A Narala N Vyas G . *In vivo* fate and activity of second- versus third-generation CD19-specific CAR-T cells in B cell non-Hodgkin’s lymphomas. Mol Ther. (2018) 26:2727–37. doi: 10.1016/j.ymthe.2018.09.009. PMID: 30309819 PMC6277484

[98] Locke FL Miklos DB Tees MT Li A Truppel-Hartmann A Flinn IW . CRC-403: A phase 1/2 study of bbT369, a dual targeting CAR T-cell drug product with a gene edit, in relapsed and/or refractory B-cell non-Hodgkin lymphoma (NHL). J Clin Oncol. (2022) 40:TPS7580–TPS7580. doi: 10.1200/JCO.2022.40.16_suppl.TPS7580. PMID: 40980460

[99] Dahiya S Ulrickson M Yared J Reagan P Voorhees T Reshef R . A phase 1 study of KITE-753 or KITE-363 in patients with relapsed/refractory B-cell lymphoma: Initial safety and preliminary efficacy of KITE-753 and updated results of KITE-363. Blood. (2025) 146:265. doi: 10.1182/blood-2025-265. PMID: 40674120

[100] Ng BD Rajagopalan A Kousa AI Fischman JS Chen S Massa A . IL-18-secreting multiantigen targeting CAR T cells eliminate antigen-low myeloma in an immunocompetent mouse model. Blood. (2024) 144:171–86. doi: 10.1182/blood.2023022293. PMID: 38579288 PMC11302468

[101] Svoboda J Landsburg DJ Gerson J Nasta SD Barta SK Chong EA . Enhanced CAR T-cell therapy for lymphoma after previous failure. N Engl J Med. (2025) 392:1824–35. doi: 10.1056/NEJMoa2408771. PMID: 40334157

[102] Asmamaw Dejenie T Tiruneh G/Medhin M Dessie Terefe G Tadele Admasu F Wale Tesega W Chekol Abebe E . Current updates on generations, approvals, and clinical trials of CAR T-cell therapy. Hum Vaccin Immunother. (2022) 18:2114254. doi: 10.1080/21645515.2022.2114254. PMID: 36094837 PMC9746433

[103] Kagoya Y Tanaka S Guo T Anczurowski M Wang C-H Saso K . A novel chimeric antigen receptor containing a JAK–STAT signaling domain mediates superior antitumor effects. Nat Med. (2018) 24:352–9. doi: 10.1038/nm.4478. PMID: 29400710 PMC5839992

[104] Yuti P Sawasdee N Natungnuy K Rujirachaivej P Luangwattananun P Sujjitjoon J . Enhanced antitumor efficacy, proliferative capacity, and alleviation of T cell exhaustion by fifth-generation chimeric antigen receptor T cells targeting B cell maturation antigen in multiple myeloma. BioMed Pharmacother. (2023) 168:115691. doi: 10.1016/j.biopha.2023.115691. PMID: 37844355

[105] Yuti P Wutti-in Y Sawasdee N Kongkhla K Phanthaphol N Choomee K . Anti-CD19 chimeric antigen receptor T cells secreting anti-PD-L1 single-chain variable fragment attenuate PD-L1 mediated T cell inhibition. Int Immunopharmacol. (2022) 113:109442. doi: 10.1016/j.intimp.2022.109442. PMID: 36435066

[106] Eyquem J Mansilla-Soto J Giavridis T van der Stegen SJC Hamieh M Cunanan KM . Targeting a CAR to the TRAC locus with CRISPR/Cas9 enhances tumour rejection. Nature. (2017) 543:113–7. doi: 10.1038/nature21405. PMID: 28225754 PMC5558614

[107] Rupp LJ Schumann K Roybal KT Gate RE Ye CJ Lim WA . CRISPR/Cas9-mediated PD-1 disruption enhances anti-tumor efficacy of human chimeric antigen receptor T cells. Sci Rep. (2017) 7:737. doi: 10.1038/s41598-017-00462-8. PMID: 28389661 PMC5428439

[108] Tang N Cheng C Zhang X Qiao M Li N Mu W . TGF-β inhibition via CRISPR promotes the long-term efficacy of CAR T cells against solid tumors. JCI Insight. (2020) 5. doi: 10.1172/jci.insight.133977. PMID: 31999649 PMC7101140

[109] Andreu-Saumell I Rodriguez-Garcia A Mühlgrabner V Gimenez-Alejandre M Marzal B Castellsagué J . CAR affinity modulates the sensitivity of CAR-T cells to PD-1/PD-L1-mediated inhibition. Nat Commun. (2024) 15:3552. doi: 10.1038/s41467-024-47799-z. PMID: 38670972 PMC11053011

[110] Hu W Zi Z Jin Y Li G Shao K Cai Q . CRISPR/Cas9-mediated PD-1 disruption enhances human mesothelin-targeted CAR T cell effector functions. Cancer Immunol Immunother. (2019) 68:365–77. doi: 10.1007/s00262-018-2281-2. PMID: 30523370 PMC11028344

[111] Agarwal S Aznar MA Rech AJ Good CR Kuramitsu S Da T . Deletion of the inhibitory co-receptor CTLA-4 enhances and invigorates chimeric antigen receptor T cells. Immunity. (2023) 56:2388–2407.e9. doi: 10.1016/j.immuni.2023.09.001. PMID: 37776850 PMC10591801

[112] Kumar J Kumar R Kumar Singh A Tsakem EL Kathania M Riese MJ . Deletion of Cbl-b inhibits CD8+ T-cell exhaustion and promotes CAR T-cell function. J Immunother Cancer. (2021) 9. doi: 10.1136/jitc-2020-001688. PMID: 33462140 PMC7813298

[113] Hu B Nastoupil LJ Holmes H Hamdan A Kanate A Farooq U . A CRISPR-edited allogeneic anti-CD19 CAR-T cell therapy with a PD-1 knockout (CB-010) in patients with relapsed/refractory B cell non-Hodgkin lymphoma (r/r B-NHL): Updated phase 1 results from the ANTLER trial. J Clin Oncol. (2024) 42:7025. doi: 10.1200/JCO.2024.42.16_suppl.7025. PMID: 41735675

[114] Hu Y Zu C Zhang M Wei G Li W Fu S . Safety and efficacy of CRISPR-based non-viral PD1 locus specifically integrated anti-CD19 CAR-T cells in patients with relapsed or refractory Non-Hodgkin’s lymphoma: a first-in-human phase I study. EClinicalMedicine. (2023) 60:102010. doi: 10.1016/j.eclinm.2023.102010. PMID: 37251628 PMC10209187

[115] Liu X Zhang Y Li K Liu Y Xu J Ma J . A novel dominant-negative PD-1 armored anti-CD19 CAR T cell is safe and effective against refractory/relapsed B cell lymphoma. Transl Oncol. (2021) 14:101085. doi: 10.1016/j.tranon.2021.101085. PMID: 33813229 PMC8050776

[116] McCutcheon SR Swartz AM Brown MC Barrera A Amador CM Siklenka K . Transcriptional and epigenetic regulators of human CD8+ T cell function identified through orthogonal CRISPR screens. Nat Genet. (2023) 55(12):2211–2223. doi: 10.1038/s41588-023-01554-0, PMID: 37945901 PMC10703699

[117] Korell F Olson ML Salas-Benito D Leick MB Larson RC Bouffard A . Comparative analysis of Bcl-2 family protein overexpression in CAR T cells alone and in combination with BH3 mimetics. Sci Transl Med. (2024) 16:eadk7640. doi: 10.1126/scitranslmed.adk7640. PMID: 38838132 PMC11737343

[118] Poorebrahim M Lee H Benaoudia S Maity R Ahn S Leblay N . Bclxl prevents progressive exhaustion in BCMA CAR T cells with maintenance of high TCF1 expressing T cells. Blood. (2023) 142:454. doi: 10.1182/blood-2023-189747. PMID: 41761659

[119] Liang C Huang S Zhao Y Chen S Li Y . TOX as a potential target for immunotherapy in lymphocytic Malignancies. biomark Res. (2021) 9:20. doi: 10.1186/s40364-021-00275-y. PMID: 33743809 PMC7981945

[120] Kim K Park S Park SY Kim G Park SM Cho J-W . Single-cell transcriptome analysis reveals TOX as a promoting factor for T cell exhaustion and a predictor for anti-PD-1 responses in human cancer. Genome Med. (2020) 12:22. doi: 10.1186/s13073-020-00722-9. PMID: 32111241 PMC7048139

[121] Scott AC Dündar F Zumbo P Chandran SS Klebanoff CA Shakiba M . TOX is a critical regulator of tumour-specific T cell differentiation. Nature. (2019) 571:270–4. doi: 10.1038/s41586-019-1324-y. PMID: 31207604 PMC7698992

[122] Li F Zhang Y . Targeting NR4As, a new strategy to fine-tune CAR-T cells against solid tumors. Signal Transduct Target Ther. (2019) 4:7. doi: 10.1038/s41392-019-0041-1. PMID: 30937188 PMC6438967

[123] Yang Z-Z Grote DM Xiu B Ziesmer SC Price-Troska TL Hodge LS . TGF-β upregulates CD70 expression and induces exhaustion of effector memory T cells in B-cell non-Hodgkin’s lymphoma. Leukemia. (2014) 28:1872–84. doi: 10.1038/leu.2014.84. PMID: 24569779 PMC4145058

[124] Yang Z Fu Y-X . Inactivation of TGF-β signaling in CAR-T cells. Cell Mol Immunol. (2024) 21:309–10. doi: 10.1038/s41423-023-01123-9. PMID: 38403679 PMC10901871

[125] Chong EA Svoboda J Dwivedy Nasta S Landsburg DJ Winchell N Napier E . Sequential anti-CD19 directed chimeric antigen receptor modified T-cell therapy (CART19) and PD-1 blockade with pembrolizumab in patients with relapsed or refractory B-cell non-hodgkin lymphomas. Blood. (2018) 132:4198. doi: 10.1182/blood-2018-99-119502. PMID: 41761659

[126] Jacobson CA Westin JR Miklos DB Herrera AF Lee J Seng J . Abstract CT055: Phase 1/2 primary analysis of ZUMA-6: Axicabtagene ciloleucel (Axi-Cel) in combination With atezolizumab (Atezo) for the treatment of patients (Pts) with refractory diffuse large B cell lymphoma (DLBCL). Cancer Res. (2020) 80:CT055–5. doi: 10.1158/1538-7445.AM2020-CT055. PMID: 41680580

[127] . JCAR014 and Durvalumab in Treating Patients With Relapsed or Refractory B-cell Non-Hodgkin Lymphoma. Identifier: NCT02706405. Phase 1 study of JCAR014 in combination with durvalumab for relapsed/refractory B-cell non-Hodgkin lymphoma. Updated 2022 Aug 24.

[128] Gill S Vides V Frey NV Hexner EO Metzger S O’Brien M . Anti-CD19 CAR T cells in combination with ibrutinib for the treatment of chronic lymphocytic leukemia. Blood Adv. (2022) 6:5774–85. doi: 10.1182/bloodadvances.2022007317. PMID: 35349631 PMC9647791

[129] Jaeger U Worel N McGuirk JP Riedell PA Fleury I Du Y . Safety and efficacy of tisagenlecleucel plus pembrolizumab in patients with r/r DLBCL: phase 1b PORTIA study results. Blood Adv. (2023) 7:2283–6. doi: 10.1182/bloodadvances.2022007779. PMID: 36044388 PMC10225880

[130] A Safety and Efficacy Trial of JCAR017 Combinations in Subjects With Relapsed/Refractory B-cell Malignancies (PLATFORM). Identifier: NCT03330691. Updated 2023 Jul 12. Available from: https://clinicaltrials.gov/study/NCT03330691. No Title.

[131] Minson A Hamad N Cheah CY Tam C Blombery P Westerman D . CAR T cells and time-limited ibrutinib as treatment for relapsed/refractory mantle cell lymphoma: the phase 2 TARMAC study. Blood. (2024) 143:673–84. doi: 10.1182/blood.2023021306. PMID: 37883795

[132] Luo W Zhang Y Li C Xu J Zhuolin W Wang X . BTK inhibitor synergizes with CD19-targeted chimeric antigen receptor-T cells in patients with relapsed or refractory B-cell lymphoma: an open-label pragmatic clinical trial. Blood. (2024) 144:6546. doi: 10.1182/blood-2024-205304. PMID: 41123227 PMC12541673

[133] Cluster of Differentiation Antigen 19/22 (CD19/22) CAR T Cells (AUTO3) for the Treatment of Diffuse Large B Cell Lymphoma (ALEXANDER). Identifier: NCT03287817. Updated 2023 May 9. Available from: https://clinicaltrials.gov/study/NCT03287817.

[134] Kiyasu J Miyoshi H Hirata A Arakawa F Ichikawa A Niino D . Expression of programmed cell death ligand 1 is associated with poor overall survival in patients with diffuse large B-cell lymphoma. Blood. (2015) 126:2193–201. doi: 10.1182/blood-2015-02-629600. PMID: 26239088 PMC4635115

[135] John LB Kershaw MH Darcy PK . Blockade of PD-1 immunosuppression boosts CAR T-cell therapy. Oncoimmunology. (2013) 2:e26286. doi: 10.4161/onci.26286. PMID: 24353912 PMC3862687

[136] Hirayama AV Kimble EL Wright JH Fiorenza S Gauthier J Voutsinas JM . Timing of anti-PD-L1 antibody initiation affects efficacy/toxicity of CD19 CAR T-cell therapy for large B-cell lymphoma. Blood Adv. (2024) 8:453–67. doi: 10.1182/bloodadvances.2023011287. PMID: 37903325 PMC10837185

[137] Chong EA Alanio C Svoboda J Nasta SD Landsburg DJ Lacey SF . Pembrolizumab for B-cell lymphomas relapsing after or refractory to CD19-directed CAR T-cell therapy. Blood. (2022) 139:1026–38. doi: 10.1182/blood.2021012634. PMID: 34496014 PMC9211527

[138] ClinicalTrials.gov. An open-label, multi-center, single-arm phase 1/2 study to assess tolerability, safety and efficacy of CRC01 in adult patients with relapsed or refractory large B-cell lymphoma. ClinicalTrials.gov Identifier: NCT04836507. Bethesda (MD).

[139] Xin X Zhu X Yang Y Wang N Wang J Xu J . Efficacy of programmed cell death 1 inhibitor maintenance after chimeric antigen receptor T cells in patients with relapsed/refractory B-cell non-Hodgkin-lymphoma. Cell Oncol (Dordrecht Netherlands). (2024) 47:1425–40. doi: 10.1007/s13402-024-00940-y. PMID: 38564164 PMC12974053

[140] Alu A Lei H Han X Wei Y Wei X . BTK inhibitors in the treatment of hematological Malignancies and inflammatory diseases: mechanisms and clinical studies. J Hematol Oncol. (2022) 15:138. doi: 10.1186/s13045-022-01353-w. PMID: 36183125 PMC9526392

[141] Burger JA . Bruton tyrosine kinase inhibitors: present and future. Cancer J. (2019) 25:386–93. doi: 10.1097/PPO.0000000000000412. PMID: 31764119 PMC7083517

[142] Dubovsky JA Beckwith KA Natarajan G Woyach JA Jaglowski S Zhong Y . Ibrutinib is an irreversible molecular inhibitor of ITK driving a Th1-selective pressure in T lymphocytes. Blood. (2013) 122:2539–49. doi: 10.1182/blood-2013-06-507947. PMID: 23886836 PMC3795457

[143] Luo W Li C Wu J Tang L Wang X Zhang Y . Bruton tyrosine kinase inhibitors preserve anti-CD19 chimeric antigen receptor T-cell functionality and reprogram tumor micro-environment in B-cell lymphoma. Cytotherapy. (2023) 25:739–49. doi: 10.1016/j.jcyt.2023.03.005. PMID: 37074239

[144] Karmali R Galvez C Winter J Ma S Zhang B Xie P . Phase II, single-arm, open-label, multicenter study: Efficacy of adjunctive Bruton’s tyrosine kinase inhibitor (BTKi) zanubrutinib and chimeric antigen receptor (CART) in aggressive B-cell non-hodgkins lymphoma (aNHL). Blood. (2025) 146:1010. doi: 10.1182/blood-2025-1010. PMID: 41761659

[145] Galustian C Labarthe M-C Bartlett JB Dalgleish AG . Thalidomide-derived immunomodulatory drugs as therapeutic agents. Expert Opin Biol Ther. (2004) 4:1963–70. doi: 10.1517/14712598.4.12.1963. PMID: 15571458

[146] Gandhi AK Kang J Havens CG Conklin T Ning Y Wu L . Immunomodulatory agents lenalidomide and pomalidomide co-stimulate T cells by inducing degradation of T cell repressors Ikaros and Aiolos via modulation of the E3 ubiquitin ligase complex CRL4(CRBN.). Br J Haematol. (2014) 164:811–21. doi: 10.1111/bjh.12708. PMID: 24328678 PMC4232904

[147] Lu G Middleton RE Sun H Naniong M Ott CJ Mitsiades CS . The myeloma drug lenalidomide promotes the cereblon-dependent destruction of ikaros proteins. Science (80-). (2014) 343:305–9. doi: 10.1126/science.1244917. PMID: 24292623 PMC4070318

[148] Bansal R Reshef R . Revving the CAR - Combination strategies to enhance CAR T cell effectiveness. Blood Rev. (2021) 45:100695. doi: 10.1016/j.blre.2020.100695. PMID: 32402724

[149] LeBlanc R Hideshima T Catley LP Shringarpure R Burger R Mitsiades N . Immunomodulatory drug costimulates T cells via the B7-CD28 pathway. Blood. (2004) 103:1787–90. doi: 10.1182/blood-2003-02-0361. PMID: 14512311

[150] Luptakova K Rosenblatt J Glotzbecker B Mills H Stroopinsky D Kufe T . Lenalidomide enhances anti-myeloma cellular immunity. Cancer Immunol Immunother. (2013) 62:39–49. doi: 10.1007/s00262-012-1308-3. PMID: 22733396 PMC4098790

[151] Ramsay AG Johnson AJ Lee AM Gorgün G Le Dieu R Blum W . Chronic lymphocytic leukemia T cells show impaired immunological synapse formation that can be reversed with an immunomodulating drug. J Clin Invest. (2008) 118:2427–37. doi: 10.1172/JCI35017. PMID: 18551193 PMC2423865

[152] Loschinski R Böttcher M Stoll A Bruns H Mackensen A Mougiakakos D . IL-21 modulates memory and exhaustion phenotype of T-cells in a fatty acid oxidation-dependent manner. Oncotarget. (2018) 9:13125–38. doi: 10.18632/oncotarget.24442. PMID: 29568345 PMC5862566

[153] Tettamanti S Rotiroti MC Giordano Attianese GMP Arcangeli S Zhang R Banerjee P . Lenalidomide enhances CD23.CAR T cell therapy in chronic lymphocytic leukemia. Leuk Lymphoma. (2022) 63:1566–79. doi: 10.1080/10428194.2022.2043299. PMID: 35259043 PMC9828187

[154] Jan M Scarfò I Larson RC Walker A Schmidts A Guirguis AA . Reversible ON- and OFF-switch chimeric antigen receptors controlled by lenalidomide. Sci Transl Med. (2021) 13. doi: 10.1126/scitranslmed.abb6295. PMID: 33408186 PMC8045771

[155] Tay T Bommakanti G Jaensch E Gorthi A Karapa Reddy I Hu Y . Degradation of IKZF1 prevents epigenetic progression of T cell exhaustion in an antigen-specific assay. Cell Rep Med. (2024) 5:101804. doi: 10.1016/j.xcrm.2024.101804. PMID: 39486420 PMC11604474

[156] Van Oekelen O Amatangelo M Guo M Upadhyaya B Cribbs AP Kelly G . Iberdomide increases innate and adaptive immune cell subsets in the bone marrow of patients with relapsed/refractory multiple myeloma. Cell Rep Med. (2024) 5:101584. doi: 10.1016/j.xcrm.2024.101584. PMID: 38776911 PMC11228551

[157] Chiu H Zhao J Ortiz Estevez M Hagner PR Gandhi AK . Mezigdomide reverses T-cell exhaustion through degradation of Aiolos/Ikaros and reinvigoration of cytokine production pathways. Blood. (2023) 142:335. doi: 10.1182/blood-2023-189445. PMID: 41761659

[158] Akbari B Ghahri-Saremi N Soltantoyeh T Hadjati J Ghassemi S Mirzaei HR . Epigenetic strategies to boost CAR T cell therapy. Mol Ther. (2021) 29:2640–59. doi: 10.1016/j.ymthe.2021.08.003. PMID: 34365035 PMC8417511

[159] Pauken KE Sammons MA Odorizzi PM Manne S Godec J Khan O . Epigenetic stability of exhausted T cells limits durability of reinvigoration by PD-1 blockade. Science. (2016) 354:1160–5. doi: 10.1126/science.aaf2807. PMID: 27789795 PMC5484795

[160] Youngblood B Hale JS Kissick HT Ahn E Xu X Wieland A . Effector CD8 T cells dedifferentiate into long-lived memory cells. Nature. (2017) 552:404–9. doi: 10.1038/nature25144. PMID: 29236683 PMC5965677

[161] Wang Y Tong C Dai H Wu Z Han X Guo Y . Low-dose decitabine priming endows CAR T cells with enhanced and persistent antitumour potential via epigenetic reprogramming. Nat Commun. (2021) 12:409. doi: 10.1038/s41467-020-20696-x. PMID: 33462245 PMC7814040

[162] You L Han Q Zhu L Zhu Y Bao C Yang C . Decitabine-mediated epigenetic reprograming enhances anti-leukemia efficacy of CD123-targeted chimeric antigen receptor T-cells. Front Immunol. (2020) 11:1787. doi: 10.3389/fimmu.2020.01787. PMID: 32973749 PMC7461863

[163] Li S Xue L Wang M Qiang P Xu H Zhang X . Decitabine enhances cytotoxic effect of T cells with an anti-CD19 chimeric antigen receptor in treatment of lymphoma. Onco Targets Ther. (2019) 12:5627–38. doi: 10.2147/OTT.S198567. PMID: 31372000 PMC6635897

[164] Porazzi P Nason S Yang Z Carturan A Ghilardi G Guruprasad P . EZH1/EZH2 inhibition enhances adoptive T cell immunotherapy against multiple cancer models. Cancer Cell. (2025) 43:537–551.e7. doi: 10.1016/j.ccell.2025.01.013. PMID: 39983725 PMC13312562

[165] Zhu M Han Y Gu T Wang R Si X Kong D . Class I HDAC inhibitors enhance antitumor efficacy and persistence of CAR-T cells by activation of the Wnt pathway. Cell Rep. (2024) 43:114065. doi: 10.1016/j.celrep.2024.114065. PMID: 38578828

[166] Kong W Dimitri A Wang W Jung I-Y Ott CJ Fasolino M . BET bromodomain protein inhibition reverses chimeric antigen receptor extinction and reinvigorates exhausted T cells in chronic lymphocytic leukemia. J Clin Invest. (2021) 131. doi: 10.1172/JCI145459. PMID: 34396987 PMC8363276

[167] Utzschneider DT Charmoy M Chennupati V Pousse L Ferreira DP Calderon-Copete S . T cell factor 1-expressing memory-like CD8+ T cells sustain the immune response to chronic viral infections. Immunity. (2016) 45:415–27. doi: 10.1016/j.immuni.2016.07.021. PMID: 27533016

[168] Wu T Ji Y Moseman EA Xu HC Manglani M Kirby M . The TCF1-Bcl6 axis counteracts type I interferon to repress exhaustion and maintain T cell stemness. Sci Immunol. (2016) 1. doi: 10.1126/sciimmunol.aai8593. PMID: 28018990 PMC5179228

[169] Fruman DA Chiu H Hopkins BD Bagrodia S Cantley LC Abraham RT . The PI3K pathway in human disease. Cell. (2017) 170:605–35. doi: 10.1016/j.cell.2017.07.029. PMID: 28802037 PMC5726441

[170] Patel K Danilov AV Pagel JM . Duvelisib for CLL/SLL and follicular non-Hodgkin lymphoma. Blood. (2019) 134:1573–7. doi: 10.1182/blood.2019001795. PMID: 31554637 PMC9635582

[171] Funk CR Wang S Chen KZ Waller A Sharma A Edgar CL . PI3Kδ/γ inhibition promotes human CART cell epigenetic and metabolic reprogramming to enhance antitumor cytotoxicity. Blood. (2022) 139:523–37. doi: 10.1182/blood.2021011597. PMID: 35084470 PMC8796652

[172] Zhang H Hu Y Shao M Teng X Jiang P Wang X . Dasatinib enhances anti-leukemia efficacy of chimeric antigen receptor T cells by inhibiting cell differentiation and exhaustion. J Hematol Oncol. (2021) 14:113. doi: 10.1186/s13045-021-01117-y. PMID: 34289897 PMC8293573

[173] Wang X Wong CW Urak R Mardiros A Budde LE Chang W-C . CMVpp65 vaccine enhances the antitumor efficacy of adoptively transferred CD19-redirected CMV-specific T cells. Clin Cancer Res. (2015) 21:2993–3002. doi: 10.1158/1078-0432.CCR-14-2920. PMID: 25838392 PMC4489991

[174] Wang X Diamond DJ Forman SJ Nakamura R . Development of CMV-CD19 bi-specific CAR T cells with post-infusion *in vivo* boost using an anti-CMV vaccine. Int J Hematol. (2021) 114:544–53. doi: 10.1007/s12185-021-03215-6. PMID: 34561840 PMC8475363

[175] . Genetically Modified T-cells (CMV-Specific CD19-CAR T-cells) Plus a Vaccine (CMV-MVA Triplex) Following Stem Cell Transplantation for the Treatment of Intermediate or High Grade B-cell Non-Hodgkin Lymphoma [Internet]. ClinicalTrials.gov. Identifier NCT054.

[176] . Genetically Modified T-cells (CMV-Specific CD19-CAR T-cells) plus a Vaccine (CMV-MVA Triplex) for the Treatment of Intermediate or High Grade B-Cell Non-Hodgkin Lymphoma [Internet]. ClinicalTrials.gov. NCT05801913; 2023 Sep 29 [cited 2026 Feb 19]. Availab.

[177] Wang F Cheng F Zheng F . Stem cell like memory T cells: A new paradigm in cancer immunotherapy. Clin Immunol. (2022) 241:109078. doi: 10.1016/j.clim.2022.109078. PMID: 35840054

[178] Arcangeli S Bove C Mezzanotte C Camisa B Falcone L Manfredi F . CAR T cell manufacturing from naive/stem memory T lymphocytes enhances antitumor responses while curtailing cytokine release syndrome. J Clin Invest. (2022) 132. doi: 10.1172/JCI150807. PMID: 35503659 PMC9197529

[179] Fraietta JA Lacey SF Orlando EJ Pruteanu-Malinici I Gohil M Lundh S . Determinants of response and resistance to CD19 chimeric antigen receptor (CAR) T cell therapy of chronic lymphocytic leukemia. Nat Med. (2018) 24:563–71. doi: 10.1038/s41591-018-0010-1. PMID: 29713085 PMC6117613

[180] Singh N Perazzelli J Grupp SA Barrett DM . Early memory phenotypes drive T cell proliferation in patients with pediatric Malignancies. Sci Transl Med. (2016) 8:320ra3. doi: 10.1126/scitranslmed.aad5222. PMID: 26738796

[181] Dreyzin A Shao L Cai Y Han KL Prochazkova M Gertz M . Immunophenotype of CAR T cells and apheresis products predicts response in CD22 CAR T cell trial for B cell acute lymphoblastic leukemia. Mol Ther. (2025) 33(7):3360–3374. doi: 10.1016/j.ymthe.2025.03.019. PMID: 40087865 PMC12265959

[182] Meyran D Zhu JJ Butler J Tantalo D MacDonald S Nguyen TN . TSTEM-like CAR-T cells exhibit improved persistence and tumor control compared with conventional CAR-T cells in preclinical models. Sci Transl Med. (2023) 15:eabk1900. doi: 10.1126/scitranslmed.abk1900. PMID: 37018415

[183] Sabatino M Hu J Sommariva M Gautam S Fellowes V Hocker JD . Generation of clinical-grade CD19-specific CAR-modified CD8+ memory stem cells for the treatment of human B-cell Malignancies. Blood. (2016) 128:519–28. doi: 10.1182/blood-2015-11-683847. PMID: 27226436 PMC4965906

